# Influence of a transient spark plasma discharge on producing high molecular masses of chemical products from l-cysteine

**DOI:** 10.1038/s41598-023-28736-4

**Published:** 2023-02-04

**Authors:** Masume Farhadi, Farshad Sohbatzadeh

**Affiliations:** 1 Department of Atomic and Molecular Physics, Faculty of Science, University of Mazandaran, Babolsar, Iran; 2 Plasma Technology Research Core, Faculty of Science, University of Mazandaran, Babolsar, Iran

**Keywords:** Techniques and instrumentation, Physics, Chemical physics

## Abstract

Cold atmospheric pressure plasmas are considered a forthcoming method in many research areas. Plasma modification of biomolecules has received much attention in addition to plasma-treated biomaterials. Hence, in this work, we operated a transient spark plasma (TSP) discharge to study its effect on the l-cysteine chemical structure. the TSP was configured in a pin-to-ring electrode arrangement and flowed by Ar gas. We also investigated the effect of two chemicals; dimethyl sulfoxide (DMSO) and hydrogen peroxide (H_2_O_2_) by the bubbling method to show how they can change the creation of new chemical bioproducts. Ultraviolet–Visible absorption spectroscopy, Fourier transform infrared spectroscopy and Liquid chromatography–mass spectroscopy were used to investigate any changes in chemical bonds of cysteine structure and to depict the generation of new biomolecules. Based on the displayed results plasma-generated reactive species had a great role in the chemical structure of the cysteine. Entering DMSO and H_2_O_2_ into the plasma caused the creation of new products and the heaviest biomolecule was produced by the simultaneous addition of DMSO and H_2_O_2_. The results also predicted that some chemical products and amino acids with a higher value molecular masse produced from the polymerization process of cysteine solution. The strong oxidation process is responsible for the heavy chemical compounds.

## Introduction

The unique ability of cold atmospheric pressure plasma to produce a wide variety of reactive species in many fields, especially plasma medicine, has been considered by many researchers. Bacteria and virus inactivation^1,2^, wound healing^3,4^, skin diseases^5,6^, and different types of cancer diseases^7–10^ are among the topics of interest treated with cold atmospheric pressure plasma. So far, a variety of plasma experimental configurations^11,12^ of the atmospheric pressure plasma jet (APPJ)^13–16^ and dielectric barrier discharge (DBD)^17–19^ have been designed and enhanced to achieve a specific goal. Changing various parameters such as voltage, current, frequency, discharge gap, and type of feeding gas, can play an important role in the formation of the amount and type of reactive species. In studying plasma medicine applications, researchers focus on biological systems. They have aimed to understand the interactions between biological samples and plasma using both simulation and experimental methods^20–22^. Organic systems such as proteins -known as complex biological systems- are under consideration by different plasma discharges. Since the amount and type of reactive species produced by plasma are very effective in treatment, scientists have studied the effect of different plasma configurations on amino acids, which are the main components of proteins. Back in 2014, Takai et al.^23^ studied the effect of a plasma jet on 20 amino acids and reported the changes on 14 amino acid side chains. In 2016, Zhou et al.^24^ upgraded the plasma jet system to a higher number of micro-plasma jets to show how plasma affects the protein's structures. Some years later, Wende et al. and Sremacki et al. used respectively a kINPen plasma jet^25^ and an RF plasma jet coupled with an aerosol system^26^ to investigate plasma–liquid interaction processes and their effect on cysteine amino acid. In addition, Lackmann et al. utilized two plasma sources to show results of chemical properties are different for each plasma source^27^. In 2014, a dielectric barrier discharge (DBD) plasma device was designed to investigate several mechanisms of the decomposed products from valine amino acid by Li et al.^28^. Also, other researchers showed that factors such as treatment time and solution concentration can affect the modification's quality^29^. Interestingly, sulfur-containing amino acids are considered a good target. It seems they undergo chemical modifications by plasma treatment more than others. As mentioned earlier, the most important feature of cold atmospheric pressure plasmas is being able to create highly reactive oxygen and nitrogen species (RONS) that stay near room temperature. Hence, they are suitable for biological system modifications. It is noticeable that interaction between plasma and aqueous medium is essential for many applications, especially biological systems. Living organisms contain water and that’s why studying plasma-liquid interaction is crucial^30–32^. In this way, scientists have focused on plasma-activated water (PAW)^33,34^. The treatment above or underneath of water surface by exposure to plasma^35,36^ converts water to an active medium including many reactive species. The interaction of plasma-derived radicals and particles with water molecules results in various chemical reactions. In fact, by trapping energetic species and particles coming from the plasma phase to the aqueous liquid, many new gas–liquid interface chemical reactions form, and then it causes to create many other reactive particles which are dissolving in water^37,38^. These reactive species may include reactive oxygen or nitrogen species such as liquid phase species (H_2_O_2_, NO_2_^–^, NO_3_^–^, ^·^OH, ONOOH, ONOO^–^) and (NO, NO_2_, O_3_, atomic O, NO, NO_2_, N_2_O, HNO_2_, HNO_3_, O_2_^–^, _1_O^2^)^39–43^. Among different types of cold atmospheric pressure plasmas, transient spark plasma discharge (TSP) is very useful due to its high electron density. TSP discharges are known as DC-driven self-pulsing with repetition frequency between 1 and 10 kHz and typically short-duration current pulses (10–100 ns)^44^. This type of plasma discharge consists of a large number of streamers with an electric field of nearly 200 kV/cm in their heads that can be transferred into short spark current pulses. This feature of TSP discharges allows ionization and effective chemical processes to be performed easily^45,46^.

Cold atmospheric pressure plasmas especially in medical areas are approaching implements for controlling different kinds of cancers^47,48^, and wounds^49^, alongside the great effects on cellular signaling processes and biomolecules' chemical modifications^50^. In addition to the use of cold plasmas in bacteria inactivation, cell death in damaged tissues, biomolecule degradation; its effect on cellular activity, and improving the chemical structures of proteins and amino acids have been highly regarded. As mentioned above, many scientists investigated the effect of cold plasmas on different amino acids as main components of proteins and observed results indicating chemical changes in amino acids' structures through attaching new bonds^51,52^ under plasma treatments. Therefore, for improving and extending polymerization processes from amino acids under plasma treatments, optimizing different plasma parameters should be more investigated.

This study is aimed to survey the impact of TSP on cysteine amino acid structure. Producing high molecular masses from monomers by consideration of various parameters is investigated. Lack of cysteine in our body can lead to some disorders such as impaired antioxidant defenses, decreased ability to metabolize drugs or toxic compounds, depressed immune functions, psychosis, and homocystinuria. Moreover, it is been currently used in some drugs, or *N*-acetyl-l-cysteine amino acid -a modified cysteine amino acid- is now available in the market as a drug. Hence, we predicted that plasma-activated cysteine can be of great interest in the fields of biomedicine and drug delivery.

The generation of new biomolecules with different m/z values is directly dependent on plasma-derived species amount. For this purpose, we designed a TSP setup that includes a pin-to-ring electrode to create a dense plasma. However, the degradation process is common during plasma treatment, we focused on adding new chemical bonds to enhance the polymerization processes. Applying chemical materials such as hydrogen peroxide (H_2_O_2_)—for its capability to create reactive radicals—and dimethyl sulfoxide (DMSO)—for containing sulfur atoms- to the plasma was also investigated to show how additives to plasma can change the production of chemical biomolecules. By adding these materials, we aimed to produce a dense plasma containing more reactive species that can be very effective in the polymerization processes. A high volume of RONS generated in the gas, liquid, and gas–liquid interface caused the formation of different types of new molecules with larger molecular masses. The vapor of these materials was injected into the system by the bubbling method and resulted in producing new chemical macromolecules. Furthermore, under the over-oxidation process and high treatment time, the production rate of reactive species increased, which can help the polymerization process and the creation of new chemical bioproducts. We aimed to control the type of plasma-produced biomolecules so that we can create combinations of several amino acids to create a long chain through the oxidation process from cysteine to make a complete peptide chain. Also, we want to use plasma-activated drugs containing amino acids in the biological fields in the next studies. This study has generally provided fundamental data in this field and many other studies and experiments need to be done to achieve peptides with TSP treatment**.**

## Materials and methods

### Experimental setup

**Figure 1:**
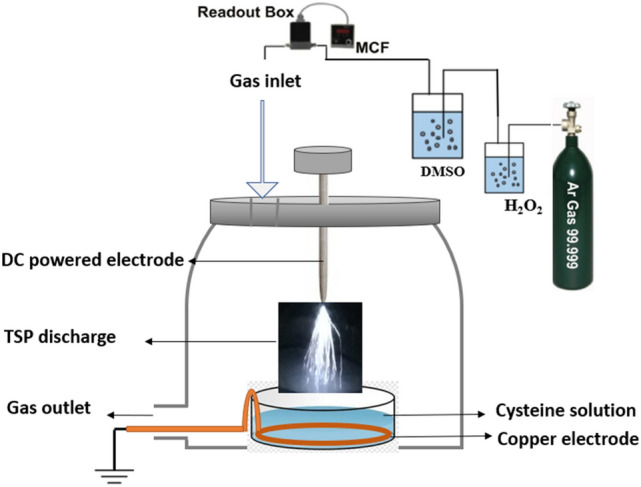
A schematic diagram of TSP discharge.

A schematic of the experimental setup is depicted in Fig. 1. This plasma source consists of two electrodes: a copper ring electrode with a diameter of approximately 4 cm and a tungsten needle electrode with a 0.8 mm diameter and a flat tip. The needle electrode was supplied with a DC power supply and its distance was adjustable with a screw. The ring electrode was grounded. Our homemade DC power supply ranges from 0 to 20 kV by the fixed voltage at ~ 14 kV for Ar and ~ 9 kV for Ar + DMSO, Ar + H_2_O_2_ + DMSO, and a frequency of 300 Hz. Furthermore, we used a 10MΩ resistor in the connection path of the electrode to the power supply.

For controlling feeding gas flow a mass flow controller (Sevenstar D07-19B) and a flow readout box (Sevenstar D08-1F) were used to throw in 2 SLM of Ar gas. The distance of the needle tip to the water surface was set to ~ 2 cm. For the next step of our experiments, as shown in Fig. 1 the feeding gas goes through an H_2_O_2_ balloon and then a DMSO balloon by the flow of 5 SLM. Finally, generated plasma was directly applied to the solution containing amino acid.

### Sample preparation

We provided cysteine (l-cysteine Merc 30,089 (≥ 98.5%)) in crystalline form and using distilled water we prepared a solution of cysteine by concentration in 10 mM. For any treatment, 4 ml of the solution was exposed to plasma. Dimethyl sulfoxide (DMSO; 99.9% C_2_H_6_SO; M = 78.13 g/mol; density = 1.1 g/cm^3^) as an organosulfur compound and hydrogen peroxide (H_2_O_2_; 35%; M = 34.01 g/m; density = 1.45 g/cm^3^) were used in our experiments. Plasma exposure time was considered for 10 min. After treatments, untreated and treated samples were used for Fourier-transform infrared spectroscopy (FTIR) analysis and liquid chromatography-mass spectroscopy (LCMS/MS). Moreover, pH measurements and ultraviolet–visible (UV–Vis) analysis were done.

### Physical measurements

For physical measurements, a digital pH meter (PET-103, Atron, Germany) was used to measure the pH value. A UV–Vis spectrometer (UV-6100; M&A INSTRUMENTS INC, USA) in the 190–400 nm range of wavelength was used. For comparing the untreated sample spectra and all other plasma-treated samples after 10 min TSP treatment, FTIR analysis (Thermo, AVATAR, USA) was performed in the range of 400–4000 cm^−1^ for characterizing the functional groups of the amino acid and new products. Finally, to detect new chemical products LCMS/MS by using a mass spectrometer (an Agilent 6410 Triple-Quad, Santa Clara, CA, USA) supplied with a positive ion mode of Electrospray Ionization interface was used.

FTIR analysis (Thermo, Nicolet Nexus 870 ESP FT-IR) operating in the transmission mode was performed in the range of 400–4000 cm^−1^ by the resolution of 4 cm^−1^ for characterizing the functional groups of the amino acid and new products. To produce consistent ultrapure water quality, a Milli-Q water purification system (resistivity 18.2 MΩ) was used. The analysis was performed based on the Csl beam splitter. In addition, to detect new biochemical molecules LCMS/MS by using a mass spectrometer (an Agilent 6410 Triple-Quad, Santa Clara, CA, USA) which was supplied with a positive ion mode of Electrospray Ionization interface was used. A UHPLC system including a binary pump (Agilent Series 1200, Agilent Technologies, Santa Clara, CA, USA), an auto-sampler, and a vacuum degasser for the LC separation was used. Separation was done on a reversed-phase rapid resolution C18 analytical column (RR Zorbax Eclipse XDB-C18) via particle size, internal diameter, and length of 1.8 μm, 4.6 mm, and 50 mm, respectively. Water with 0.1% formic acid and acetonitrile was used for the A and B mobile phases, respectively. The chromatographic method carried the starting mobile phase organization (10% B) constant for 1 min, then a linear gradient to 100% B at 11 min. Finally, 100% B moved through the column for 4 min with a flow rate of 0.6 mL min^-1^. Mass Spectroscopy analysis was done by an Agilent 6410 Triple-Quad mass spectrometer (Agilent Technologies Series 1200, Santa Clara, CA, USA). The device was equipped with electrospray ionization (ESI) in positive ion mode (Nitrogen as the nebulizer and collision gas, a capillary voltage of 5000 V, gas flow and temperature: 12 L min^−1^; 325 °C, and nebulizer gas: 50 psig). In addition, Agilent Mass Hunter software was used for data processing and for obtaining maximum intensities, collision energies, and cone voltages of two MRM transitions that were optimized with a continuous flow of a standard injection.

## Results and discussion

### pH measurement and UV–Vis analysis

**Figure 2:**
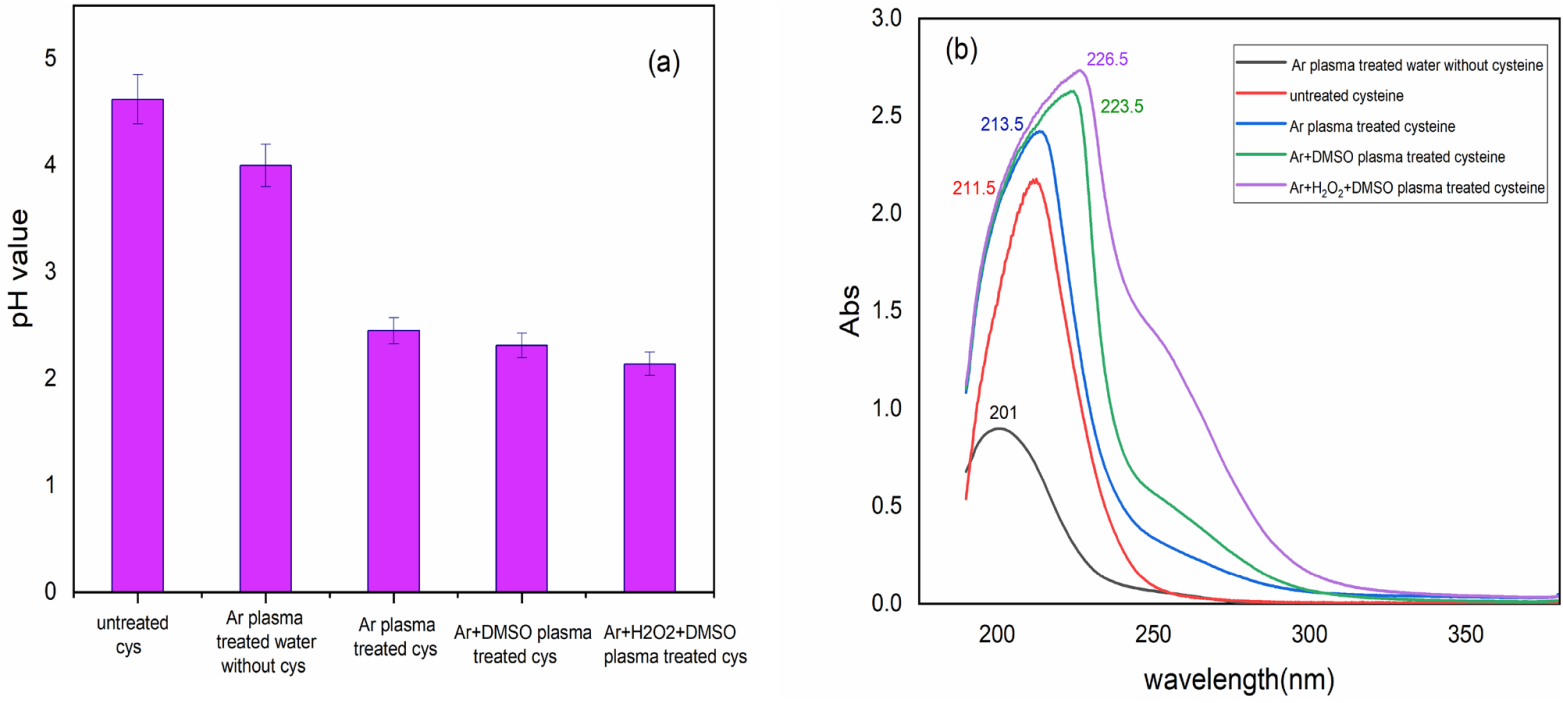
(**a**) Comparison of the pH value of untreated cysteine solution with different plasma-treated samples and (**b**) UV–Vis absorption spectra of cysteine solution and different types of TSP-treated cysteine.

The pH values of untreated cysteine solution besides other treated samples with different plasmas are shown in Fig. 2a. The pH value dropped dramatically after TSP treatments. Firstly, Ar plasma-treated water without cysteine showed a lower value of pH than untreated cysteine solution (dropped from 4.62 to 4.10). Secondly, the pH value of the cysteine solution after Ar + DMSO plasma exposure reached half of that for the untreated sample and reached pH = 2.31. Without DMSO this value became a little bit more than half (pH = 2.45). Finally, adding both H_2_O_2_ and DMSO resulted in the minimum value of pH = 2.14. This means that the solution has changed to a more acidic medium under the influence of Ar + H_2_O_2_ + DMSO plasma than other plasma-treated samples.

Figure 2b shows the comparison UV–Vis spectrum of untreated and plasma-treated cysteine solutions. According to Szili et. al^53^ UV absorbance contributes to the main long-lived reactive species such as NO_2_^–^, NO_3_^–^, and H_2_O_2_. Increased shoulder in 230–330 nm contributes to H_2_O_2_ concentration, whereas increased intensity in peak at 190–250 nm contributes to NO_2_^–^ and NO_3_^–^ concentrations. As can be seen, adding H_2_O_2_ to the plasma led to a high amount of H_2_O_2_ concentration. In addition, related peaks of NO_2_^–^ and NO_3_^–^ tolerate more intensities after different plasma treatments which were again the highest for Ar + H_2_O_2_ + DMSO plasma^54,55^. Finally, it can be seen from Fig. 2b that the absorbance of the NO_2_^–^ and NO_3_^–^, and H_2_O_2_ in Ar plasma-treated water without cysteine is less than that of the Ar plasma-treated cysteine sample. This indicates that the role of NO_2_^–^ and NO_3_^–^, and H_2_O_2_ in absorbance is minor concerning the new products of the Ar plasma-treated cysteine samples.

Interestingly, a red-shifted tendency in all cases of plasma treatment was also observed (Ar + H_2_O_2_ + DMSO > Ar + DMSO > Ar). According to Ankireddy et. Al.^56^, the cause of the red-shifted modified peak may be due to the formation of n–π* transitions. The solution decreases the energy state of plasma-derived excited electrons and the red-shifted effect increases by increasing the solution polarity. By the addition of both DMSO and H_2_O_2_ through Ar plasma, the mentioned wavelength went through a larger wavelength that shows it contains a larger number of mentioned species than others do.

### FTIR analysis

For the molecular structures and functional groups of amino acids, FTIR analysis has been considered a useful tool. We surveyed FTIR spectra changes in the range of 400–4000 cm^−1^ to show how different plasmas affect the cysteine solution. All spectra were converted from transmitted spectra to absorption spectra by using the below formula:

$$A = {\text{log}}\left( {\frac{1}{{\text{T}}}} \right)$$

**Figure 3:**
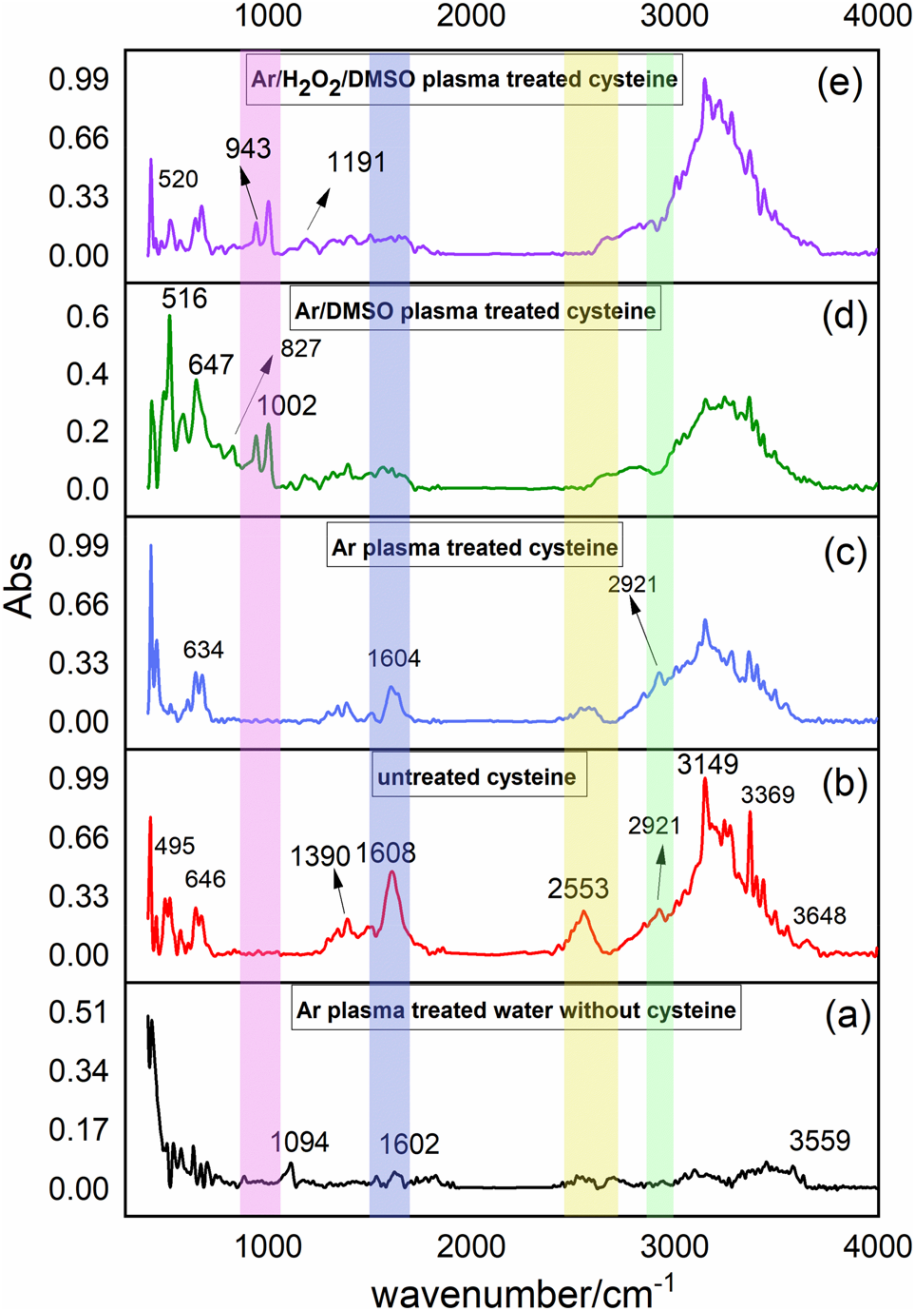
FTIR spectra of (**a**) Ar plasma-treated water without cysteine, (**b**) untreated cysteine solution and plasma-treated cysteine solution by (**c**) Ar plasma, (**d**) Ar + DMSO plasma, and (**e**) Ar + H_2_O_2_ + DMSO plasma. (Highlighted regions show appearing or disappearing bonds of (green) C–H, (yellow) S–H, (blue) COO^–^, (pink) S–S, and S–O).

Figure 3 compares the FTIR spectra of untreated and Ar plasma-treated samples with and without the presence of DMSO and H_2_O_2_. Changes in the position and the intensity of peaks have occurred in the presence of plasma for all treated samples.

In the range of 2000–4000 cm^−1^, several bonds such as O–H (3369 cm^−1^) hydroxyl group stretching vibrations in carboxylic, N–H (3243 cm^−1^) stretching vibration of amine –NH_2_ functional group of untreated cysteine solution^57^, C–H (3149 cm^−1^), vibration aliphatic C–H (2923 cm^−1^) for charged amine –NH^3+^^58^, S–H thiol group (2553 cm^−1^) thiol group were detected for untreated cysteine. The S–H bond as the characteristic peak of cysteine disappeared in plasma-treated solutions. Some mentioned bonds were seen with a slightly shifted position in the plasma-treated cases. Chemical bonds in the range of 400–1800 cm^−1^ also showed several changes. Asymmetric and symmetric stretching vibration of COO^–^ (1608 cm^−1^ and 1390 cm^−1^), These bonds also changed to 1604 cm^−1^ and high-intensity peak 1386 cm^−1^ in the Ar plasma-treated sample and decreased dramatically after adding DMSO and H_2_O_2_^59–61^. The C–S bond (595–830 cm^−1^)^62,63^ was also detected. After adding DMSO and H_2_O_2_ new peaks at (1191 cm^−1^), (1002 cm^−1^), and (943 cm^−1^) respectively related to S=O, S–O, and S–S were detected. New peaks of (516 cm^−1^) in Ar + DMSO and (520 cm^−1^) in Ar + H_2_O_2_ + DMSO were also added. These peaks contributed to the S–S bond by more intensity in Ar + DMSO plasma and its absorbance decreased after adding H_2_O_2_ in Ar + H_2_O_2_ + DMSO.

The intensity of an absorption bond depends on the polarity of the bond. So that a bond with higher polarity will show a more intense absorption bond. The intensity also depends on the number of bonds responsible for the absorption. Moreover, the dipole moment base on the chemical impact of plasma treatment of cysteine with high electron density may be the reason for the slight shift in the overall frequencies^64,65^. In other words, changes in the electron distribution and hybridization state result in the position of peaks. In FTIR, the changes in peak intensity and position usually indicate changes in molecular bonds and functional groups creating some complexes. As a result, it is confirmed that the polymerization process has occurred.

### Detection of cysteine derivatives under TSP treatment by LCMS/MS analysis

Mass Spectroscopy analysis was done by an Agilent 6410 Triple-Quad mass spectrometer (Agilent Technologies Series 1200, Santa Clara, CA, USA) and a UHPLC system was used to investigate any changes in the chemical structure of cysteine.

**Figure 4:**
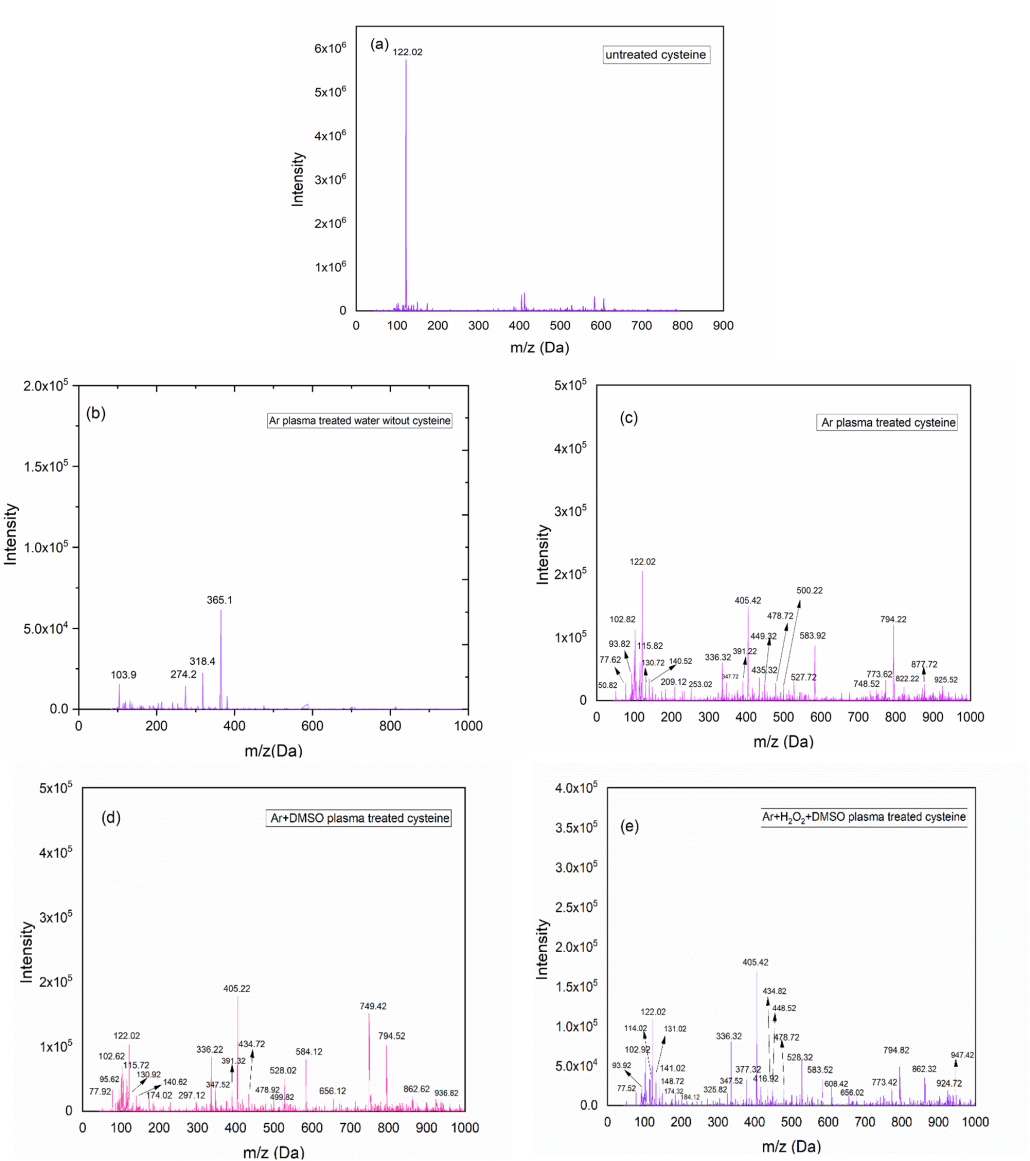
Mass Spectra of (**a**) untreated cysteine, (**b**) Ar plasma treated water without cysteine, and TSP-derived biomolecules from treated cysteine with (**c**) Ar plasma, (**d**) Ar + DMSO plasma, (**e**) Ar + H_2_O_2_ + DMSO.

**Figure 5:**
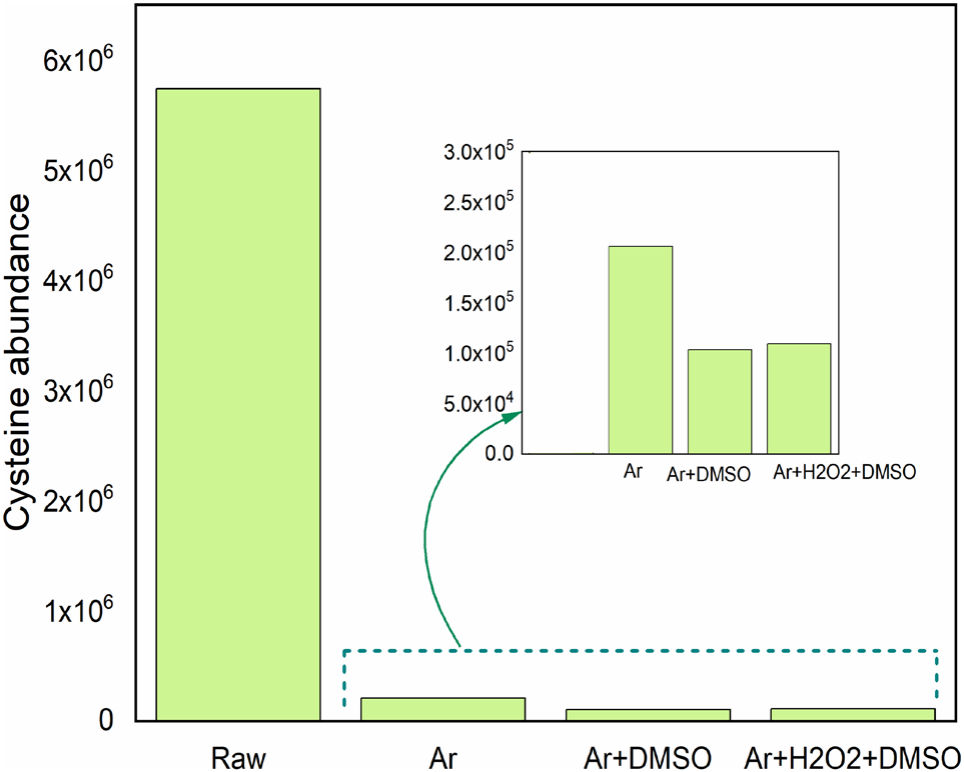
Comparison of the abundance of cysteine in the untreated sample with the other three plasmas with Ar as feeding gas.

Figure 4 shows the mass spectra of untreated and treated samples. As can be seen in all treated cysteine (Fig. 4c–e), many new biomolecules are detected compared to untreated ones (Fig. 4a). The abundance of cysteine based on the bar chart in Fig. 5 followed Raw > Ar > Ar + H_2_O_2_ + DMSO > Ar + DMSO. It means Ar + DMSO plasma had the most impact on converting cysteine to other molecules. However, other types of plasmas had a great influence on producing new biomolecules.

**Figure 6:**
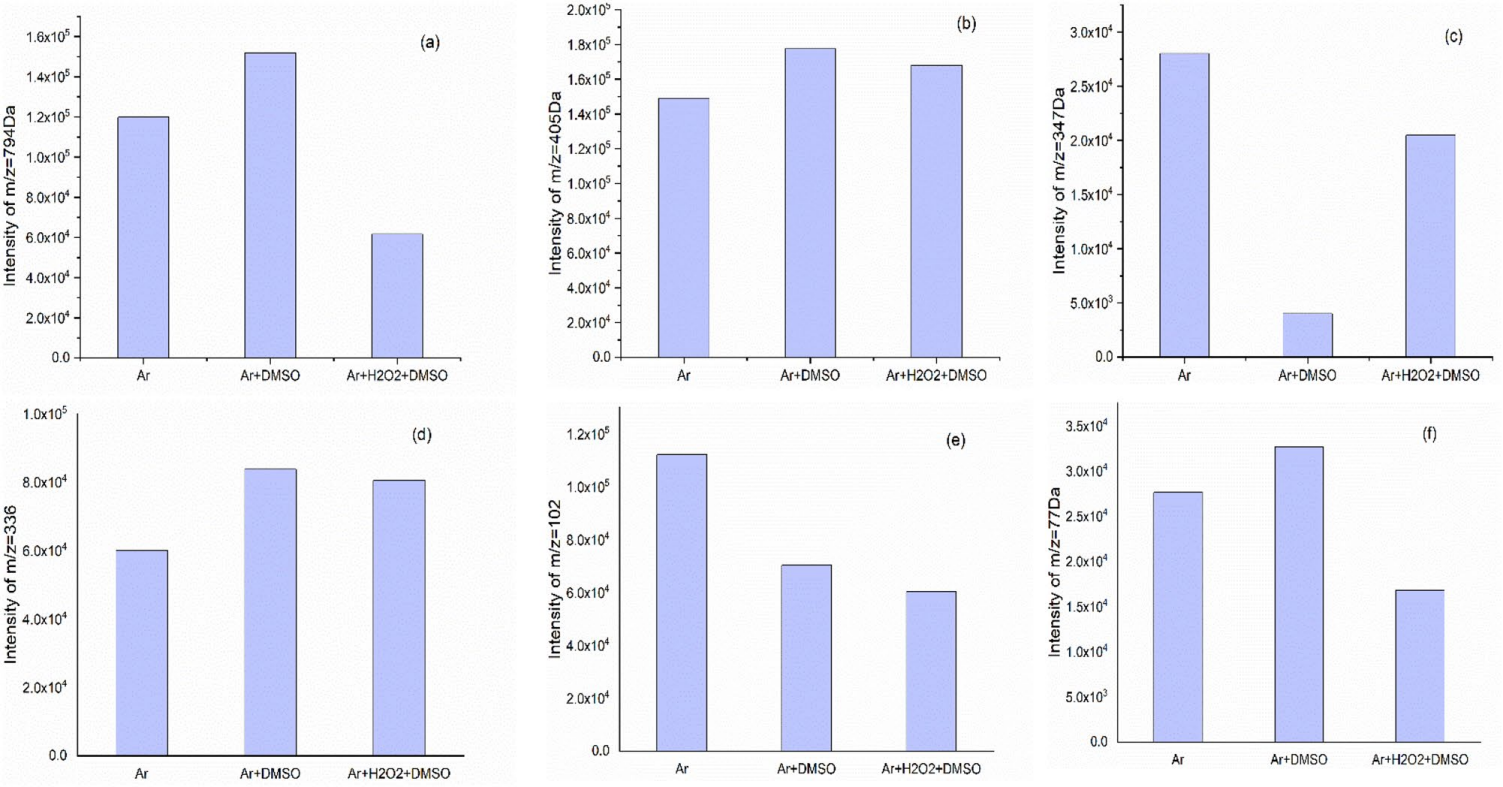
Abundance comparing of m/z = (**a**)794 Da, (**b**)405 Da, (**c**)347 Da, (**d**)336 Da, (**e**)102 Da, (**f**)77 Da.

These peaks are recorded in Table 1 based on their m/z values and their predicted chemical structures are available in the supplementary information. Although some peaks with the same molecular mass have been observed in all three cases of treated cysteine, the abundance of these products is different in each of them (Fig. 6). Furthermore, some unique chemical products are generated as a result of each plasma treatment that was the most in the Ar + H_2_O_2_ + DMSO plasma treatment. Besides the chaining process to create macromolecules, each treatment led to degradation and the minimum m/z value was seen for Ar plasma treatment (m/z = 50 Da). For the peaks that were observed in all three treatments, they showed the highest abundance as a result of the Ar + DMSO treatment except for (m/z = 347 Da and 102 Da). Interestingly, the Ar + H_2_O_2_ + DMSO plasma treatment led to generating the biggest product nearly to 1KDa (m/z = 947 Da; C_25_H_38_N_8_O_15_S_8_) which may be due to the more amount of RONS species.

**Table 1:** The m/z [M–H]^+^ values of major cysteine derivatives induced under different plasma treatments.

Ar plasma		Ar + DMSO plasma		Ar + H_2_O_2_ + DMSO plasma	
Compound	(m/z)	Compound	(m/z)	Compound	(m/z)
H_2_OS	50.82	C_2_H_4_O_3_	77.92	C_2_H_4_O_3_	77.52
C_2_H_4_O_3_	77.62	CH_5_NO_2_S	95.62	CH_3_NO_2_S	93.92
CH_3_NO_2_S	93.82	C_4_H_7_NO_2_	102.62	C_4_H_7_NO_2_	102.82
C_4_H_7_NO_2_	102.82	C_5_H_9_NO_2_	115.72	C_5_H_7_NO_2_	114.02
C_5_H_9_NO_2_	115.82	C_3_H_7_NO_2_S	122.02	C_3_H_7_NO_2_S	122.02
C_3_H_7_NO_2_S	122.02	C_5_H_7_NO_3_	130.92	C_7_H_14_O_2_	131.02
C_5_H_7_NO_3_	130.72	C_3_H_9_NO_3_S	140.62	C_3_H_8_O_4_S	141.02
C_3_H_9_NO_3_S	140.52	C_3_H_11_NO_5_S	174.02	C_5_H_9_NO_4_	148.72
C_5_H_8_N_2_O_3_S_2_	209.12	C_8_H_12_N_2_O_4_S_3_	297.12	C_3_H_11_NO_5_S	174.32
C_7_H_12_N_2_O_4_S_2_	253.02	C_6_H_13_N_3_O_9_S_2_	336.22	C_4_H_9_NO_3_S_2_	184.12
C_6_H_13_N_3_O_9_S_2_	336.32	C_8_H_14_N_2_O_9_S_2_	347.52	C_6_H_16_N_2_O_9_S_2_	325.82
C_8_H_14_N_2_O_9_S_2_	347.72	C_10_H_18_N_2_O_8_S_3_	391.32	C_6_H_13_N_3_O_9_S_2_	336.32
C_10_H_18_N_2_O_8_S_3_	391.22	C_9_H_16_N_4_O_8_S_3_	405.22	C_8_H_14_N_2_O_9_S_2_	347.52
C_9_H_16_N_4_O_8_S_3_	405.42	C_11_H_19_N_3_O_9_S_3_	434.72	C_9_H_16_N_2_O_8_S_3_	377.32
C_10_H_18_N_4_O_9_S_3_	435.32	C_10_H_14_N_4_O_10_S_4_	478.92	C_9_H_16_N_4_O_8_S_3_	405.42
C_9_H_11_N_3_O_12_S_3_	449.32	C_12_H_22_N_2_O_11_S_4_	499.82	C_11_H_17_N_3_O_8_S_3_	416.92
C_10_H_14_N_4_O_10_S_4_	478.72	C_12_H_21_N_3_O_12_S_4_	528.02	C_9_H_14_N_4_O_10_S_3_	434.82
C_11_H_21_N_3_O_11_S_4_	500.22	C_9_H_20_N_4_O_17_S_4_	584.12	C_9_H_13_N_5_O_10_S_3_	448.52
C_13_H_22_N_2_O_12_S_4_	527.72	C_11_H_20_N_4_O_16_S_6_	656.12	C_10_H_14_N_4_O_10_S_4_	478.72
C_9_H_21_N_5_O_16_S_4_	583.92	C_18_H_29_N_5_O_15_S_6_	749.42	C_12_H_21_N_3_O_12_S_4_	528.32
C_18_H_29_N_5_O_15_S_6_	748.52	C_18_H_31_N_7_O_16_S_6_	794.52	C_9_H_21_N_5_O_16_S_4_	583.52
C_24_H_36_O_16_S_6_	773.62	C_18_H_35_N_7_O_18_S_7_	862.62	C_9_H_16_N_6_O_17_S_4_	608.42
C_18_H_31_N_7_O_16_S_6_	794.22	C_23_H_33_N_7_O_19_S_7_	936.82	C_11_H_20_N_4_O_16_S_6_	656.02
C_20_H_35_N_7_O_16_S_6_	822.22			C_24_H_36_O_16_S_6_	773.42
C_18_H_36_N_8_O_18_S_7_	877.72			C_18_H_31_N_7_O_16_S_6_	794.82
C_23_H_36_N_6_O_19_S_7_	925.52			C_18_H_35_N_7_O_18_S_7_	862.32
				C_24_H_37_N_5_O_19_S_7_	924.72
				C_25_H_38_N_8_O_15_S_8_	947.42

**Figure 7:**
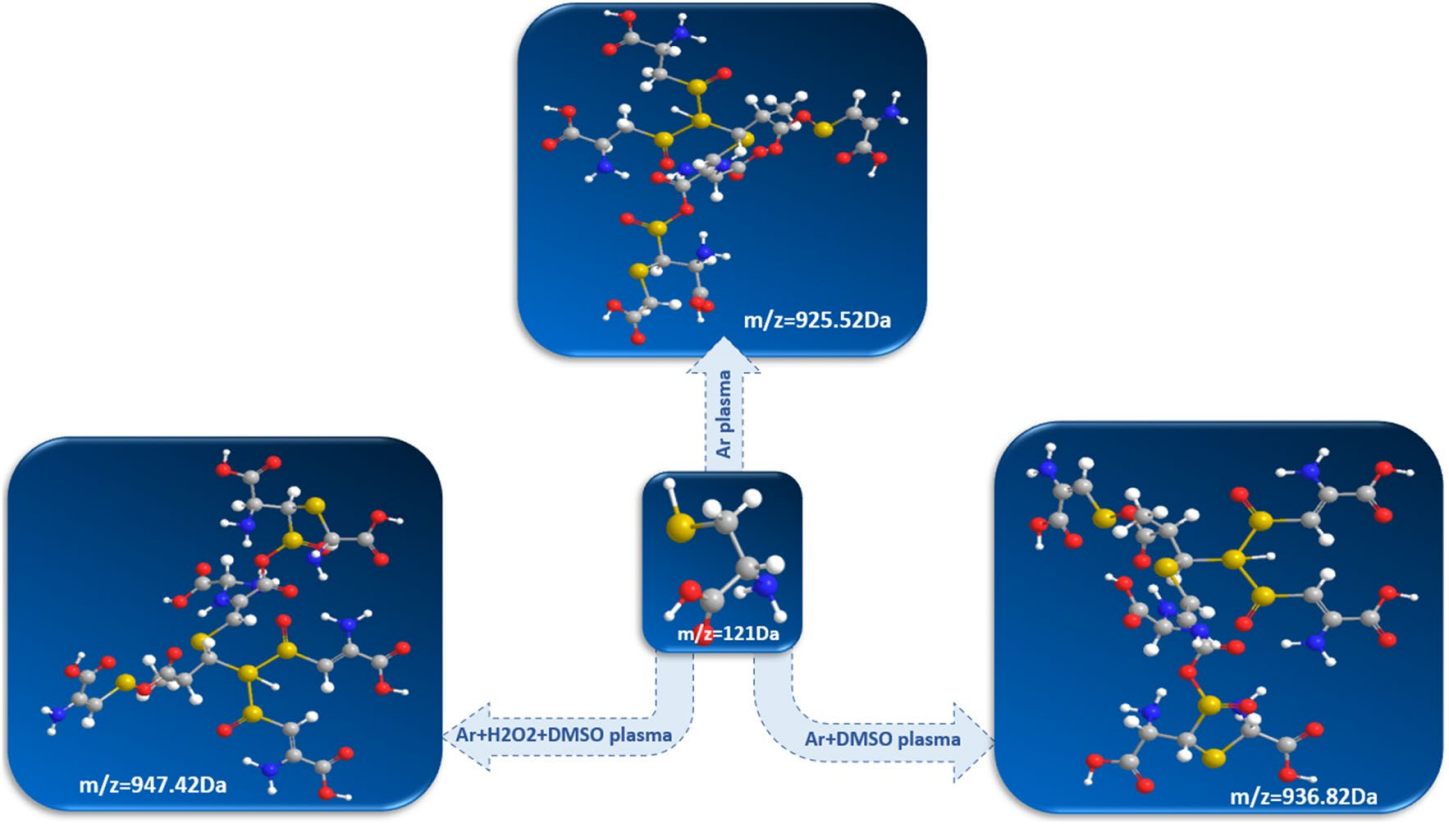
3D structures of the heaviest TSP-derived chemical products.

The 3D structures of the heaviest chemical products (m/z = 925 Da; C_23_H_36_N_6_O_19_S_7_, m/z = 936 Da; C_23_H_33_N_7_O_19_S_7_ and m/z = 947 Da; C_25_H_38_N_8_O_15_S_8_,) respectively derived from Ar, Ar + DMSO and Ar + H_2_O_2_ + DMSO plasmas, are shown in Fig. 7.

Full information and predicted chemical structures of the produced compounds are compiled in the supplementary information file, and as an example, you can see some of the larger compounds produced by different plasma treatments in Table 2.

**Table 2:** Characteristics of some large TSP-derived compounds.

Plasma	Chemical formula	[M–H] ^+^(m/z)	Chemical structure/systematic name
Ar Ar + H_2_O_2_ + DMSO	C_9_H_21_N_5_O_16_S_4_	583.92	(((2-carboxy-1-((2-carboxy-2-hydrazineylethyl) sulfonyl)-2-hydrazineylethyl) sulfonyl) sulfonyl) (dihydroxy(oxo)-l6-sulfaneyl) (hydroxy)alanine
Ar + DMSO Ar + H_2_O_2_ + DMSO	C_11_H_20_N_4_O_16_S_6_	656.12	N-(3-(((amino(carboxy)methyl)sulfinyl)oxy)-2-(hydroxyamino)-3-oxopropyl)-C3-((((2-carboxy-2-(hydroxyamino)ethyl)sulfinyl)thio)sulfinyl)-C3-(hydrosulfinylsulfinyl)-N-hydroxyalanine
Ar + DMSO	C_18_H_29_N_5_O_15_S_6_	749.42	(2Z,7Z)-2,11,16-triamino-10-(1,3-bis(2-amino-2-carboxyethyl)-1,3-dioxo-1l4,2l4,3l4-trisulfaneyl)-7-hydroxy-6,12-dioxo-5,13-dioxa-4,9,14-trithiaheptadeca-2,7-dienedioic acid
Ar@Ar + H_2_O_2_ + DMSO	C_24_H_36_O_16_S_6_	773.62	(2E,7E)-10-(1,3-bis(2-carboxypropyl)-1,1,3,3-tetraoxo-1l6,2l4,3l6-trisulfaneyl)-2,7,11,16-tetramethyl-6,12-dioxo-5,13-dioxa-4,9,14-trithiaheptadeca-2,7-dienedioic acid
Ar Ar + DMSO Ar + H_2_O_2_ + DMSO	C_18_H_31_N_7_O_16_S_6_	794.22	(2Z,7Z)-7,11,16-triamino-10-(1,3-bis(2-amino-2-carboxyethyl)-1,1,3,3-tetraoxo-1l6,2l4,3l6-trisulfaneyl)-2-hydrazineyl-6,12-dioxo-5,13-dioxa-4,9,14-trithiaheptadeca-2,7-dienedioic acid
Ar + DMSO Ar + H_2_O_2_ + DMSO	C_18_H_35_N_7_O_18_S_7_	862.62	2-amino-11-(1,3-bis(2-amino-2-carboxyethyl)-1,1,3,3-tetraoxo-1l6,2l4,3l6-trisulfaneyl)-12-hydrazineyl-8,17-bis(hydroxyamino)-7,13-dioxo-6,14-dioxa-4,5,10,15-tetrathiaoctadecanedioic acid
Ar	C_18_H_36_N_8_O_18_S_7_	877.72	11-(1,3-bis(2-amino-2-carboxyethyl)-1,1,3,3-tetraoxo-1l6,2l4,3l6-trisulfaneyl)-8,12-dihydrazineyl-2,17-bis(hydroxyamino)-7,13-dioxo-6,14-dioxa-4,5,10,15-tetrathiaoctadecanedioic acid
Ar	C_23_H_36_N_6_O_19_S_7_	925.52	(4Z,12E)-2,13-diamino-4-((((2-amino-2-carboxy-1-((carboxymethyl)thio)ethyl)sulfinyl)oxy)carbonyl)-7-(1,3-bis(2-amino-2-carboxyethyl)-1,3-dioxo-1l4,2l4,3l4-trisulfaneyl)-8-methyl-9-oxo-10-oxa-6,11-dithia-3-azatetradeca-4,12-dienedioic acid
Ar + DMSO	C_23_H_33_N_7_O_19_S_7_	936.82	(4Z,12E)-2,13-diamino-4-((((2-amino-1-((amino(carboxy)methyl)thio)-2-carboxyethyl)sulfinyl)oxy)carbonyl)-7-(1,3-bis((Z)-2-amino-2-carboxyvinyl)-1,3-dioxo-1l4,2l4,3l4-trisulfaneyl)-8-methyl-9-oxo-10-oxa-6,11-dithia-3-azatetradeca-4,12-dienedioic acid
Ar + H_2_O_2_ + DMSO	C_25_H_38_N_8_O_15_S_8_	947.42	(1Z,6Z,11Z,21Z)-2,7,12,17,22,27-hexaamino-28-((3-amino-1-(1-aminopropyl)-4-oxo-3,4-dihydro-1H-1l4,2l4-dithiin-2-yl)oxy)-3,8,13,18,23,28-hexaoxo-4,9,14,19,24-pentaoxa-5,10,15,20,25-pentathiaoctacosa-1,6,11,21-tetraene-1-sulfinic acid

However, the mechanism of plasma, chemical reactions in water, and their interface reactions are more complex than it seems, according to previous studies oxidative stress mainly contributed to the increase in the level of RONS and high reactive radicals such as ^·^H and ^·^OH. Several chemical reaction pathways (degradation and polymerization) take place using such radicals^66^. Mechanisms happening in PAW are not only by the hydrogen atom and OH radical but also by hydrated electrons (e^–^_aq_). Back in the 1980s, studies showed that e^–^_aq_ is produced in water by photons (UV photoexcitation) irradiations with energies more than 6 eV^67,68^. At this energy, the formation of e^–^_aq_ is due to H atom transfer however additional formation is possible at higher energy levels. e^–^_aq_ derived from generated secondary electrons in water as a result of ionization. These electrons have a great impact on chemistry and biological reactions. See some related reactions below:

1 $${\text{h}}\upsilon \, + {\text{ H}}_{{2}} {\text{O}} \to {\text{OH }} + {\text{ H}}$$

2 $${\text{H }} + {\text{ H}}_{{2}} {\text{O }} \to {\text{ H}}_{{3}} {\text{O}}$$

3 $${\text{H}}_{{3}} {\text{O }} \to {\text{ H}}_{{3}} {\text{O}}^{ + } + {\text{ e}}^{ - }_{{{\text{aq}}}}$$

4 $${\text{2H}}_{{2}} {\text{O }} + {\text{h}}\upsilon \to {\text{ H}}_{{3}} {\text{O}}^{ + } + {\text{ OH }} + {\text{ e}}^{ - }_{{{\text{aq}}}}$$

where H_3_O is known as a “Rydberg radical” in which an electron is weakly bound to H_3_O^+^.

5 $${\text{H}}_{{2}} {\text{O }} \to {\text{ H }} + {\text{ OH}}$$

6 $${\text{e}}^{ - }_{{{\text{aq}}}} + {\text{ H}}_{{2}} {\text{O }} \to {\text{ H }} + {\text{ OH}}^{ - }$$

7 $${\text{e}}^{ - }_{{{\text{aq}}}} + {\text{ H}}^{ + } \left( {{\text{or H}}_{{3}} {\text{O}}^{ + } } \right) \, \to {\text{ H }}({\text{or H }} + {\text{ H}}_{{2}} {\text{O}})$$

For activated water, the primary ionization occurs (reaction 1), and a hydrogen atom and OH radical generate as a result of excitation (reaction 2). Reactions (3) and (4) are two important recombination processes in which e^–^_aq_ converts to the hydrogen atom. Reducing species formed in reaction (1) in their reactions with hydrogen peroxide (8). Finally, an exciting water molecule is formed.

8 $${\text{H }} + {\text{ H}}_{{2}} {\text{O}}_{{2}} \to {\text{ H}}_{{2}} {\text{O }} + {\text{ OH}}$$

9 $${\text{H}}_{{2}} {\text{O}}^{ + } + {\text{ e}}^{ - }_{{{\text{aq}}}} \to {\text{ H}}_{{2}} {\text{O}}^{*}$$

Plasma-generated electrons, and various short and long-lived radicals when reaching the solution many mixed reactions occur. As a result of these reactions many other radicals and reactive species generate. Moreover, many RONS in PAW is generated by such long (nitrates (NO_3_^–^), nitrites (NO_2_^–^), hydrogen peroxide (H_2_O_2_), and ozone (O_3_)) and short-lived species (hydroxyl radicals (OH^·^), nitric oxide (NO^·^), superoxide (O_2_^–^), peroxynitrite (OONO_2_^–^) and peroxynitrites (ONOO^–^))^69^. Several factors such as the power supply of plasma, applied voltage, discharge regimes, treatment time, type of feeding gas and its flow, and type and configuration of electrodes, besides the solution volume and composition, and the distance between the electrode and surface of the solution, are important in the efficiency of experiments^70^. Ar plasma without any molecular gas has high-energy electrons and (V)UV photons^71,72^. The carbonyl group is high in the Ar plasma, which causes carbonylation (oxidation process). In addition, hydroxyl radicals, and atomic and singlet oxygen are shown to be effective on thiol groups in proteins under plasma in situ or ex-situ treatment. The utilization of Ar gas, H_2_O_2,_ and DMSO as the precursors, leads to the production of diverse primary species which will subsequently provide for the generation of many secondary species. With dry feed Ar gas plasma, ^·^OH as strong radicals that can oxidize cysteine may be produced both in the gas phase and in the plasma-sample interface^73–75^. The energy of the S–H bond is only 3.6 eV and is a good target to be attacked by reactive ^·^OH. H_2_O_2_ is another major ROS generated by the cold plasma (mostly as a result of recombination of OH radicals) which has a great role in cell redox signaling pathways. Singlet oxygen is also a reactive oxygen species that play an important role to oxidize cysteine. In Ar plasma, the reaction between ROS such as ^·^OH radicals and amino acid may result in the formation of hydrogen radicals. In a more acidic solution (Ar + H_2_O_2_ + DMSO in our study) NO_2_^–^ can be easily protonated and degraded to NO_2_, NO, and H_2_O. RNS is very important from two points of view:1- generation of amino acids and nitrogen compounds; and 2- as a signaling molecule for controlling metabolism. A combination of NO_2_^–^ with H_2_O_2_ and O_3_ produces nitrate (NO_3_^–^)^76^.

During the deprotonation process of hydroperoxyl radical (OOH), another important radical called superoxide (O_2_^–^) is generated in PAW. NO radical can help produce other RONS by attacking the amino acid during the oxidation process. ONOOH and ONOO can be produced through reactions:

10 $${\text{NO}}_{{2}}^{ - } + {\text{ H}}_{{2}} {\text{O}}_{{2}} + {\text{ H}}^{ + } \to {\text{ ONOOH }} + {\text{ H}}_{{2}} {\text{O}}$$

11 $${\text{O}}_{{2}}^{ - } + {\text{ NO }} \to {\text{ NO}}_{{3}}^{ - } + {\text{ ONOO}}^{ - }$$

**Figure 8:**
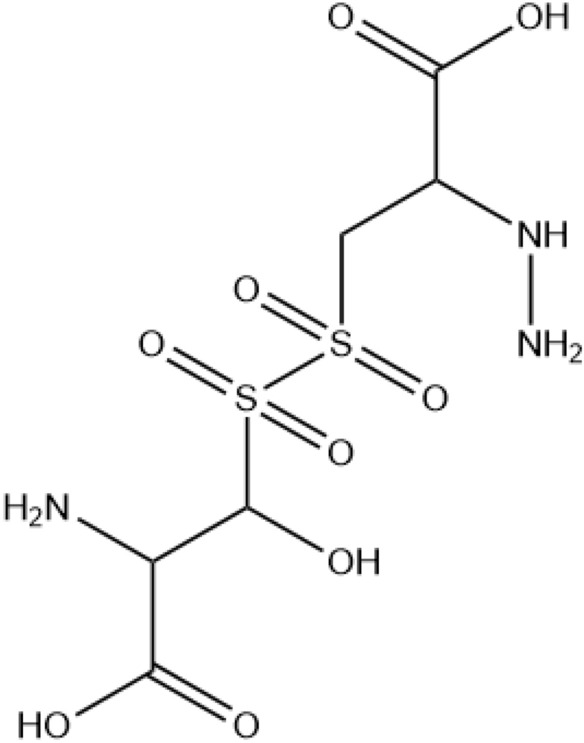
Predicted chemical structure of a biomolecule derived from all plasmas (C_6_H_13_N_3_O_9_S_2_, m/z = 336 Da).

As a result of these reactions, some bonds break while many new bonds form to create numerous bioproducts which have been detected in mass spectra. Breaking the C–S bond and creating a C–C and C=C bond (according to FTIR) was predicted and the amino group (NH_2_) was attacked with NO radical from plasma to add a new group of NH_2_. The predicted pathways of some chemical compounds were shown in our previous study. For example, a single Cysteine molecule when attacked by OH radical, and then by O_3_ Sulfinic acid can be produced. Further exposure treatment time can oxidize this product to the other structure (Tables S2, S3) by further OH attacking with a molecular weight of (C_3_H_11_NO_5_S; m/z = 174 Da). One common product during plasma treatment is attaching two cysteines whit H-abstraction of them. As mentioned earlier, because of the long treatment time and according to Zhou et.al^24^, cystine can convert to other new products under an over-oxidation process. The final product of this pathway under further exposure contains serine molecules in its structure by (C_6_H_13_N_3_O_9_S_2_, m/z = 336 Da) from all plasma treatments (see Fig. 8). By attaching three cysteine-(–SH) molecules and new C–N, S–S, and S=O bonds, a compound (C_11_H_21_N_3_O_11_S_4_; m/z = 500 Da) contains alanine amino acid was observed from Ar plasma treatment. Some products by (m/z = 583, 608, 656 Da) contain alanine amino acids in their structures. For example, (C_9_H_21_N_5_O_16_S_4_; m/z = 583 Da) shows more cysteine molecules are joined together, each with new OH bonds and an amino group. In addition, all sulfur atoms were bombarded with plasma-derived radicals (e.g., O_3_, OH, H_2_O_2_, O_2_). However, this is the first step in this direction and a lot of research needs to be done in this regard, this process brings us closer to our goal of making a complete chain of the peptide.

The amount of ROS decreases after reacting with the amino acid information of NO_2_^–^ in the media. The formation of ions such as NO_2_^–^ and NO_3_^–^ causes decreasing the value of the pH and consequently the formation of reactive nitrogen compounds. Thus, nitrogen-related radicals can alter amino acids. NO groups can attach to the thiol residues of amino acids or even to amino groups^61,69^. Interestingly, O(^3^P) and O_2_(^1^Δ_g_) as gas phase-derived species exhibited a vital role in forming products and are involved in different modifications of biomolecules. By adding DMSO to plasma, the reactive sulfur radical releases and participate in many other reactions. Moreover, some other reactive sulfur species (RSS)^77–79^ generate in solution (containing cysteine) cause the formation of chemical reactions to produce many new biomolecules.

12 $${\text{R}}{-}{\text{SH }} +^{ \cdot } {\text{OH}} \to {\text{R}}{-}{\text{S}}^{ \cdot } + {\text{ H}}_{{2}} {\text{O}}$$

13 $${\text{R}}{-}{\text{S}}^{ \cdot } +^{ \cdot } {\text{OH}} \to {\text{R}}{-}{\text{S}}{-}{\text{OH}}$$

14 $${\text{R}}{-}{\text{S}}^{ \cdot } + {\text{ O}}_{{2}} (^{{1}} \Delta_{{\text{g}}} ) \, \to {\text{R}}{-}{\text{SO}}{-}{\text{O}}^{ \cdot }$$

15 $${\text{R}}{-}{\text{SH }} + {\text{ O}}_{{2}} (^{{1}} \Delta_{{\text{g}}} ) \, \to {\text{ H}}^{ + } + {\text{ R}}{-}{\text{SO}}{-}{\text{O}}^{ - }$$

16 $${\text{R}}{-}{\text{S}}^{ \cdot } + {\text{ O }}\left( {^{{3}} {\text{P}}} \right) \, \to {\text{R}}{-}{\text{S}}{-}{\text{O}}^{ \cdot }$$

17 $${\text{R}}{-}{\text{S}}{-}{\text{OO}}^{ \cdot } {\text{/R}}{-}{\text{S}}{-}{\text{O}}^{ \cdot } + {\text{ H}}_{{2}} {\text{O }} \to^{ \cdot } {\text{OH }} + {\text{ R}}{-}{\text{S}}{-}{\text{OH}}$$

18 $${\text{R}}{-}{\text{SO}}{-}{\text{OH }} + \, [{\text{O}},{\text{ O}}_{{2}} (^{{1}} \Delta_{{\text{g}}} )] \, \to {\text{ H}}^{ + } + {\text{ R}}{-}{\text{SO}}_{{2}} {-}{\text{O}}^{ - }$$

19 $${\text{R}}{-}{\text{S}}{-}{\text{OH }} + \, [{\text{O}}_{{2}} (^{{1}} \Delta_{{\text{g}}} ),{\text{ O }}\left( {^{{3}} {\text{P}}} \right),{\text{ OH}}] \, \to {\text{ R}}{-}{\text{SO}}_{{2}} {-}{\text{OH}},{\text{ R}}{-}{\text{SO}}{-}{\text{OH}}$$

The below reaction shows the great effect of ^·^OH radical in the degradation process of DMSO:

20 $$\left( {{\text{CH}}_{{3}} } \right)_{{2}} {\text{SO }} +^{ \cdot } {\text{OH }} \to {\text{ HCHO }} + {\text{ CH}}_{{3}} {\text{SO}}_{{2}}^{ - } + {\text{ HO}}_{{2}}^{ \cdot } + {\text{H}}^{ + }$$

Under the over-oxidation process, many other relations occur as follows:

21 $${\text{CH}}_{{3}} {\text{SO}}_{{2}}^{ - } +^{ \cdot } {\text{OH }} \to {\text{ CH}}_{{3}} {\text{SO}}_{{2}}^{ \cdot } + {\text{ OH}}^{ - }$$

22 $${\text{CH}}_{{3}} {\text{SO}}_{{2}}^{ \cdot } + {\text{ O}}_{{2}} \to {\text{ CH}}_{{3}} {\text{S}}\left( {\text{O}} \right)_{{2}} {\text{O}}_{{2}}^{ \cdot }$$

23 $${\text{CH}}_{{3}} {\text{S}}\left( {\text{O}} \right)_{{2}} {\text{O}}_{{2}}^{ \cdot } + {\text{ CH}}_{{3}} {\text{SO}}_{{2}}^{ - } \to {\text{ CH}}_{{3}} {\text{S}}\left( {\text{O}} \right)_{{2}} {\text{O}}^{ \cdot } + {\text{ CH}}_{{3}} {\text{SO}}_{{3}}^{ - }$$

24 $${\text{CH}}_{{3}} {\text{S}}\left( {\text{O}} \right)_{{2}} {\text{O}}^{ \cdot } + {\text{ CH}}_{{3}} {\text{SO}}_{{2}}^{ - } \to {\text{ CH}}_{{3}} {\text{SO}}_{{3}}^{ - } + {\text{ CH}}_{{3}} {\text{SO}}_{{2}}^{ \cdot }$$

25 $${\text{CH}}_{{3}} {\text{S}}\left( {\text{O}} \right)_{{2}} {\text{O}}_{{2}}^{ \cdot } + {\text{ CH}}_{{3}} {\text{SO}}_{{2}}^{ - } \to {\text{ CH}}_{{3}} {\text{SO}}_{{3}}^{ - } + {\text{ HCHO }} + {\text{HSO}}_{{3}}^{ - } + {\text{ HO}}_{{2}}^{ \cdot } + {\text{H}}^{ + }$$

26 $${\text{HCHO }} +^{ \cdot } {\text{OH }} + {\text{ O}}_{{2}} \to {\text{ HCOO}}^{ - } + {\text{ HO}}_{{2}}^{ \cdot } + {\text{H}}^{ + }$$

27 $${\text{HCOO}}^{ - } + {\text{ HO}}_{{2}}^{ \cdot } + {\text{H}}^{ + } \to {\text{ HO}}_{{2}}^{ \cdot } + {\text{H}}_{{2}} {\text{O }} + {\text{H}}^{ + }$$

**Figure 9:**
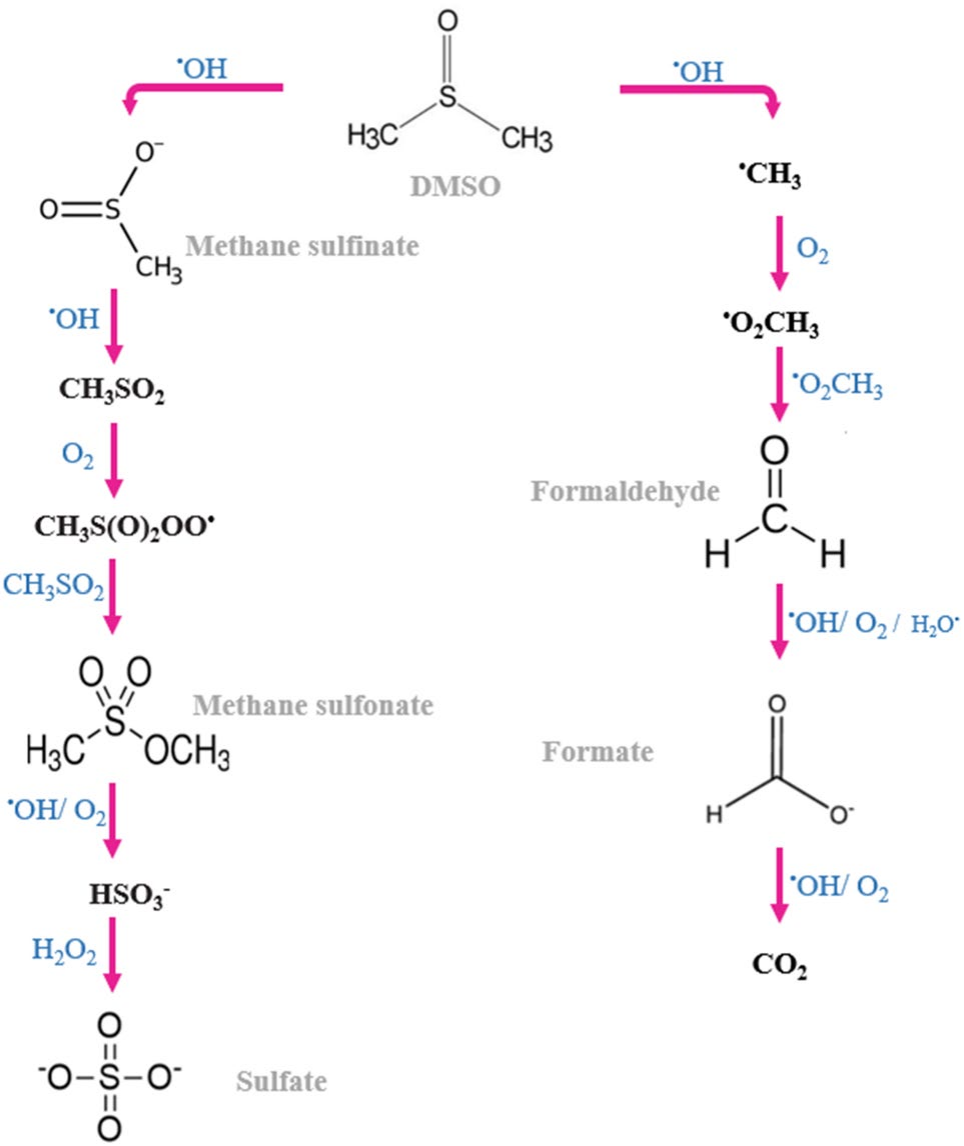
Predicted pathways of DMSO degradation processes^81^.

As a result of DMSO and ^·^OH radical interaction, two essential products (Sulfur dioxide (SO_2_) and dimethyl sulfone (C_2_H_6_SO_2_)) in the gas phase are produced. Other important factors in the DMSO oxidation process are UV radiation and H_2_O_2_^80,81^. Adding H_2_O_2_ makes it possible to generate other reactive radicals such as HOO^·^ and ^·^OH^82,83^. This leads to the acceleration of the degradation processes of DMSO. The predicted pathway of DMSO degradation is shown in Fig. 9 which is important to generate chemical products for the polymerization process from cysteine.

**Figure 10:**
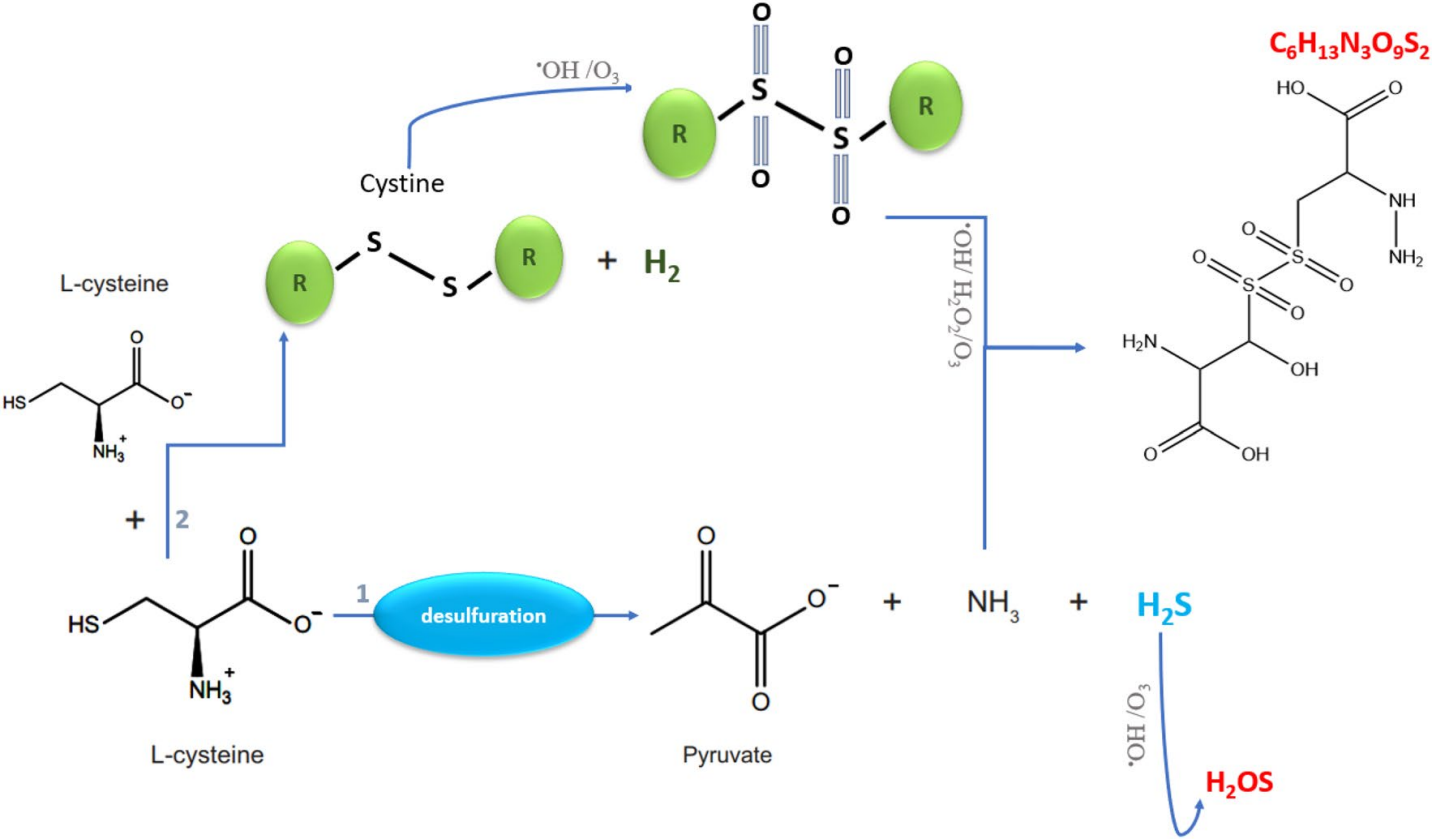
Predicted pathway of two bioproducts: (**1**) H_2_OS (m/z = 50 Da) and (**2**) C_6_H_13_N_3_O_9_S_2_ (m/z = 336 Da).

Hydrogen sulfide (H_2_S) can be generated in cells via an enzymatic or non-enzymatic pathway. H_2_S in the body acts as a gaseous signaling molecule that is known to inhibit Complex IV of the mitochondrial electron transport chain which effectively reduces ATP generation and biochemical activity within cells^84^. Releasing this molecule by pathway number (1) in Fig. 10 under the oxidation process can produce bioproduct by (m/z = 50 Da). The NH_3_ group, on the other hand, may contribute to other reactions in producing other products, for example through the pathway (2) (m/z = 336 Da).

As said earlier, the cleavage of a C–S bond also results in some new products. OH, radical is predicted to be a cause of weakening the C–N bond^76,85,86^. The over-oxidation process may lead to the demolishing of the S–S bond in some products:

28 $${\text{R}}{-}{\text{S}}{-}{\text{S}}{-}{\text{R }} \to^{ \cdot } {\text{R }} + {\text{ R}}{-}{\text{S}}{-}{\text{S}}^{ \cdot }$$

29 $$^{ \cdot } {\text{SH }} + {\text{ O}}_{{2}} \to {\text{ H}}^{ + } + {\text{ SO}}_{{2}}^{ \cdot - }$$

30 $${\text{SO}}_{{2}}^{ \cdot } + {\text{ O}}_{{2}} \to {\text{ O}}_{{2}}^{ \cdot - } + {\text{ SO}}_{{2}}$$

31 $${\text{SO}}_{{2}} + {\text{ H}}_{{2}} {\text{O }} \to {\text{ H}}^{ + } + {\text{ HSO}}_{{3}}^{ - }$$

32 $${\text{R}}{-}{\text{S}}{-}{\text{S}}{-}{\text{R }} + {\text{ HSO}}_{{3}}^{ - } \to {\text{ R}}{-}{\text{SH }} + {\text{ R}}{-}{\text{S}}{-}{\text{SO}}_{{3}}^{ - }$$

33 $${\text{R}}{-}{\text{SH }} + {\text{ HSO}}_{{3}} \to {\text{ H}}_{{2}} + {\text{ R}}{-}{\text{S}}{-}{\text{SO}}_{{3}}^{ - }$$

It is noticeable that (V)UV radiation derives from plasma through cleavage of the C–S bond can be responsible for the formation of alanine and further oxidation of the SH radical. In addition to serine and alanine, other molecules are produced during TSP treatments. It is understood that the type of reactive species and the time of treatment^6,87^ are very influential in the production and type of biochemical products^88,89^.

Sulfonic acid, sulfinic acid, carboxylic acid, acrylic acid, glycine, proline, and some other amino acid biomolecules were observed in some derivatives by different values of m/z from different TSP treatments. It seems that the ability of all three types of plasma in decomposition and degradation processes is almost the same.

## Conclusion

In summary, a TSP configuration has been developed to produce bioproducts with high molecular masses during the polymerization process from cysteine. Producing high molecular masses from monomers is directly dependent on plasma-derived species amount. Cysteine is under consideration as a biological model to enhance the finding of the redox activity of peptides and proteins. However, there are still concerns about the electron transfer reactions of these molecules that need further investigation; cysteine is still an important amino acid for monitoring the chemical structure, and capabilities of proteins due to its high oxidation states. Reactive species of Ar TSP discharge led to the generation of many oxidized cysteine productions. Besides, to some extent adding DMSO and H_2_O_2_ led to generating other new bioproducts. According to mass spectroscopy, distinct cysteine oxidation products were observed in the high value of molecular masses for all plasma treatments, confirming the potential biological impacts of RONS. Ar + H_2_O_2_ + DMSO greatly affected the thiol group which was consistent with FTIR analysis. The simultaneous addition of H_2_O_2_ and DMSO increased the number of new chemical products. Some Ar + DMSO-derived chemical products which were seen in others had more abundance. Therefore, the type of precursors and any additives to plasma can vary producing chemical products and their intensity. Generally, to grow the formation of biochemical compounds some considerations related to discharge parameters must be taken.

It is concluded that TPS treatment of cysteine can change its chemical structure dramatically. Besides the degradation process, polymerization processes have occurred to produce new bioproducts with higher molecular masses. Our results indicate that the chemical structure of cysteine is changed dramatically to create many other products. The over-oxidation process can produce larger biomolecules containing other amino acids. Finally, it is considered that many plasma-derived reactive species that were produced in the gas phase, liquid phase, and gas–liquid interface had a great effect in speeding up the polymerization processes.

## Supplementary Information

Supplementary Information.

## Data Availability

All chemical structures of generated biomolecules under different plasma treatments are presented in the Supplementary information tables.
