# Supplementary material for: Influence of a transient spark plasma discharge on producing high molecular masses of chemical products from l-cysteine
Source: Sci Rep. 2023 Feb 4;13:2059. doi: 10.1038/s41598-023-28736-4 (PMC9899256; doi:10.1038/s41598-023-28736-4)
Supplement: Supplementary file 1 — Supplementary Information. [file 41598_2023_28736_MOESM1_ESM.docx]

**Influence of a transient spark plasma discharge on producing high molecular masses of chemical products from L-Cysteine**

**Masume Farhadi^1^ & Farshad Sohbatzadeh^1,2^**^*^

^1^ Department of Atomic and Molecular Physics, Faculty of Science, University of Mazandaran, Babolsar, Iran

^2^ Plasma Technology Research Core, Faculty of Science, University of Mazandaran, Babolsar, Iran

^*^Corresponding Author: [f.sohbat@umz.ac.ir](mailto:f.sohbat@umz.ac.ir)

**SUPPLEMENTARY INFORMATION – Supplementary Tables**

**List of Supplementary Tables**

**Supplementary Table S1.** Summary of chemical formula, [M-H]^+^ *m/z* values, systematic name, and chemical structures of several observed biomolecules from cysteine amino acid under Ar TS plasma treatment.

**Supplementary Table S2.** Summary of chemical formula, [M-H]^+^ *m/z* values, systematic name, and chemical structures of several observed biomolecules from cysteine amino acid under Ar+DMSO TS plasma treatment.

**Supplementary Table S3.** Summary of chemical formula, [M-H]^+^ *m/z* values, systematic name, and chemical structures of several observed biomolecules from cysteine amino acid under Ar+H_2_O_2_+DMSO TS plasma treatment.

**Table S1: Biomolecules derived from Ar plasma**

|  | Formula | [M-H] ^+^(m/z) | Chemical Structure/  Systematic name |
| --- | --- | --- | --- |
| 1 | H_2_OS | 50.82 | 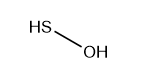  sulfanol |
| 2 | C_2_H_4_O_3_ | 77.62 | 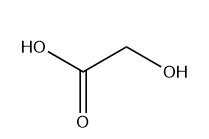  2-hydroxyacetic acid |
| 3 | CH_3_NO_2_S | 93.82 | 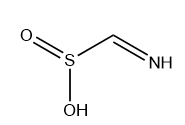  Iminomethane sulfinic acid |
| 4 | C_4_H_7_NO_2_ | 102.82 | 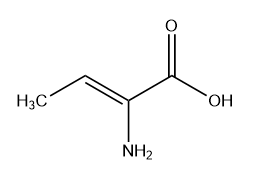  (Z)-2-aminobut-2-enoic acid |
| 5 | C_5_H_9_NO_2_ | 115.82 | 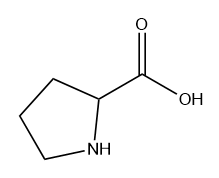  proline |
| 6 | C_3_H_7_NO_2_S | 122.02 |   cysteine |
| 7 | C_5_H_7_NO_3_ | 130.72 | 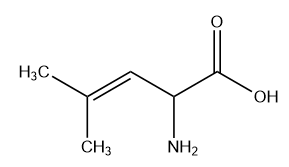  2-acetamidoacrylic acid |
| 8 | C_3_H_9_NO_3_S | 140.52 | 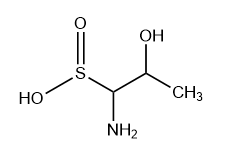  1-amino-2-hydroxypropane-1-sulfinic acid |
| 9 | C_5_H_8_N_2_O_3_S_2_ | 209.12 | 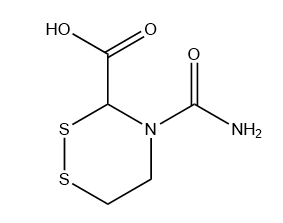  4-carbamoyl-1,2,4-dithiazinane-3-carboxylic acid |
| 10 | C_7_H_12_N_2_O_4_S_2_ | 253.02 | 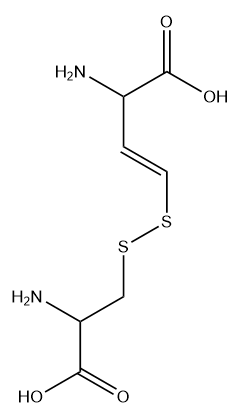  (E)-2-amino-4-((2-amino-2-carboxyethyl)disulfaneyl)but-3-enoic acid |
| 11 | C_6_H_13_N_3_O_9_S_2_ | 336.32 | 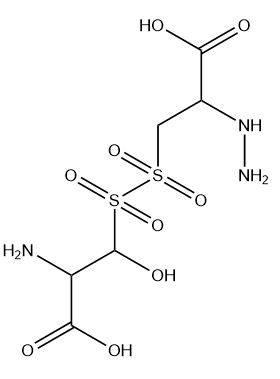  (((2-carboxy-2-hydrazineylethyl)sulfonyl)sulfonyl)serine |
| 12 | C_8_H_14_N_2_O_9_S_2_ | 347.72 | 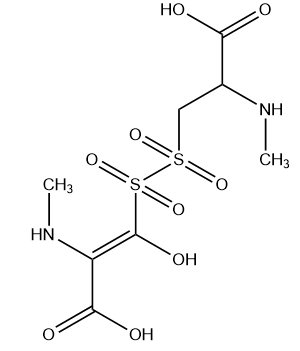  (Z)-3-(((2-carboxy-2-(methylamino)ethyl)sulfonyl)sulfonyl)-3-hydroxy-2-(methylamino)acrylic acid |
| 13 | C_10_H_18_N_2_O_8_S_3_ | 391.22 | 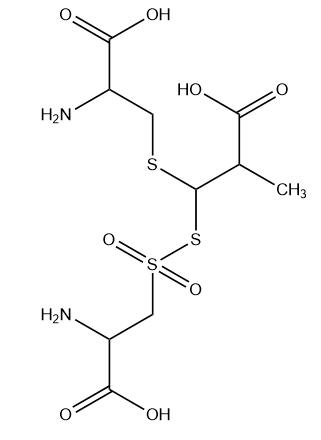  3-(((2-amino-2-carboxyethyl)sulfonyl)thio)-3-((2-amino-2-carboxyethyl)thio)-2-methylpropanoic acid |
| 14 | C_9_H_16_N_4_O_8_S_3_ | 405.42 | 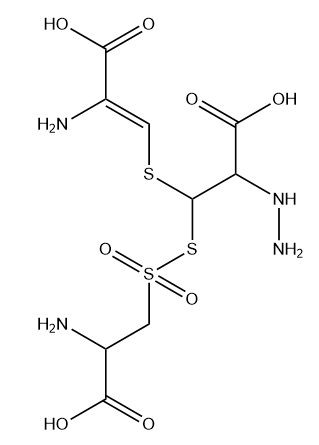  (Z)-2-amino-3-((1-(((2-amino-2-carboxyethyl)sulfonyl)thio)-2-carboxy-2-hydrazineylethyl)thio)acrylic acid |
| 15 | C_10_H_18_N_4_O_9_S_3_ | 435.32 | 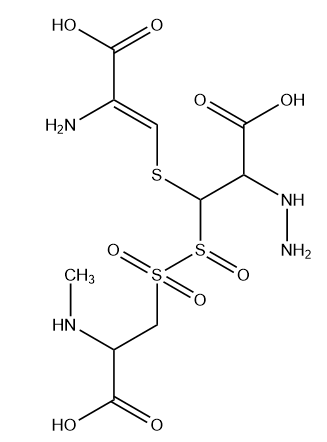  (Z)-2-amino-3-((2-carboxy-1-(((2-carboxy-2-(methylamino)ethyl)sulfonyl)sulfinyl)-2-hydrazineylethyl)thio)acrylic acid |
| 16 | C_9_H_11_N_3_O_12_S_3_ | 449.32 | 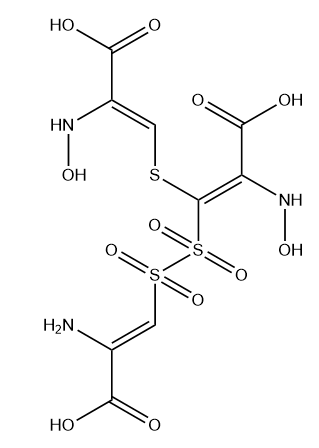  (Z)-3-((((Z)-2-amino-2-carboxyvinyl)sulfonyl)sulfonyl)-3-(((Z)-2-carboxy-2-(hydroxyamino)vinyl)thio)-2-(hydroxyamino)acrylic acid |
| 17 | C_10_H_14_N_4_O_10_S_4_ | 478.72 | 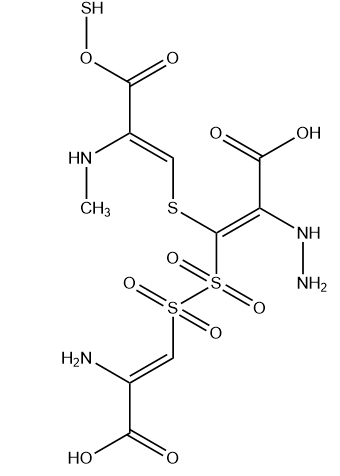  (Z)-3-((((Z)-2-amino-2-carboxyvinyl)sulfonyl)sulfonyl)-2-hydrazineyl-3-(((Z)-3-mercaptooxy-2-(methylamino)-3-oxoprop-1-en-1-yl)thio)acrylic acid |
| 18 | C_11_H_21_N_3_O_11_S_4_ | 500.22 | 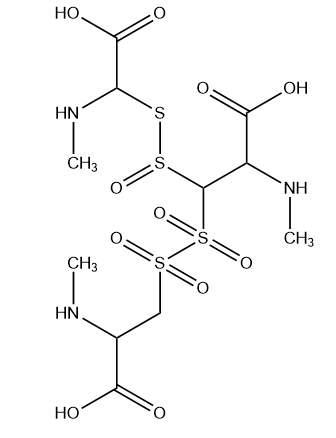  (((carboxy(methylamino)methyl)thio)sulfinyl)(((2-carboxy-2-(methylamino)ethyl)sulfonyl)sulfonyl)(methyl)alanine |
| 19 | C_13_H_22_N_2_O_12_S_4_ | 527.72 | 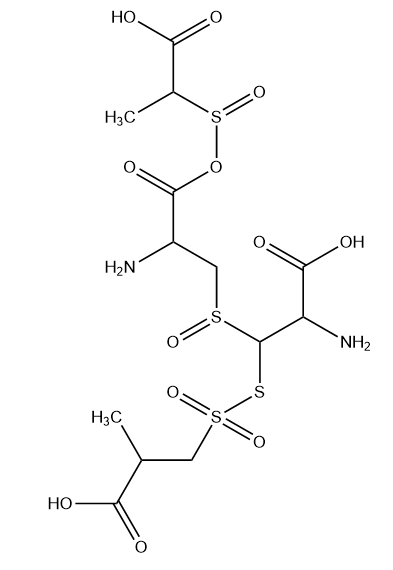  C3-((2-amino-3-(((1-carboxyethyl)sulfinyl)oxy)-3-oxopropyl)sulfinyl)-S-((2-carboxypropyl)sulfonyl)cysteine |
| 20 | C_9_H_21_N_5_O_16_S_4_ | 583.92 | 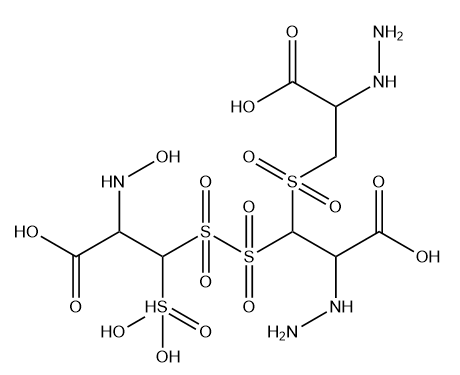  (((2-carboxy-1-((2-carboxy-2-hydrazineylethyl)sulfonyl)-2-hydrazineylethyl)sulfonyl)sulfonyl)(dihydroxy(oxo)-l6-sulfaneyl)(hydroxy)alanine |
| 21 | C_18_H_29_N_5_O_15_S_6_ | 748.52 | 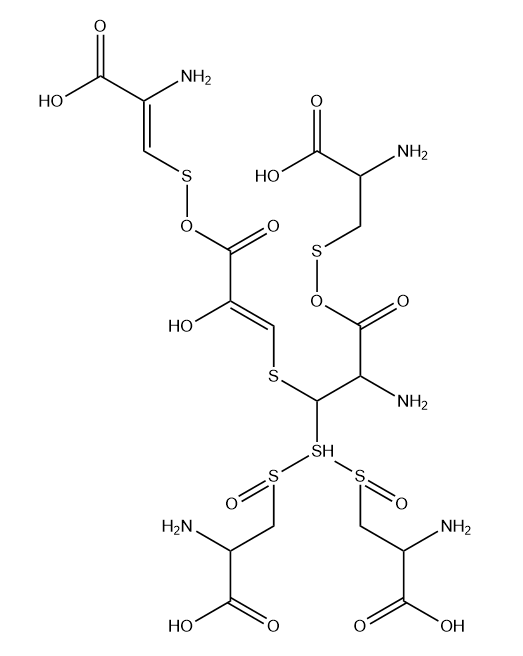  (2Z,7Z)-2,11,16-triamino-10-(1,3-bis(2-amino-2-carboxyethyl)-1,3-dioxo-1l4,2l4,3l4-trisulfaneyl)-7-hydroxy-6,12-dioxo-5,13-dioxa-4,9,14-trithiaheptadeca-2,7-dienedioic acid |
| 22 | C_24_H_36_O_16_S_6_ | 773.62 | 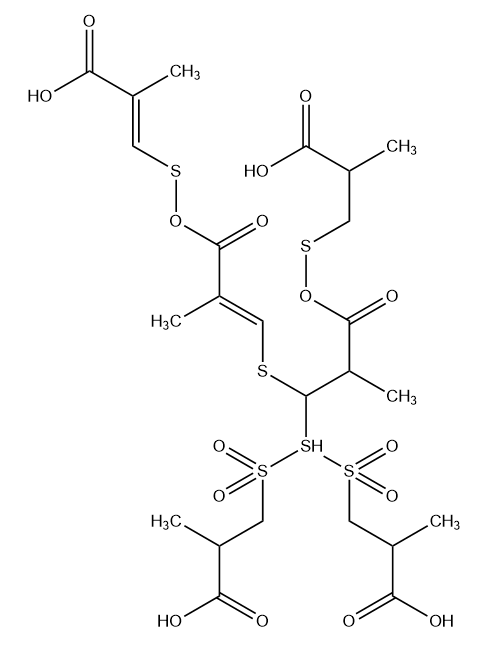  (2E,7E)-10-(1,3-bis(2-carboxypropyl)-1,1,3,3-tetraoxo-1l6,2l4,3l6-trisulfaneyl)-2,7,11,16-tetramethyl-6,12-dioxo-5,13-dioxa-4,9,14-trithiaheptadeca-2,7-dienedioic acid |
| 23 | C_18_H_31_N_7_O_16_S_6_ | 794.22 | 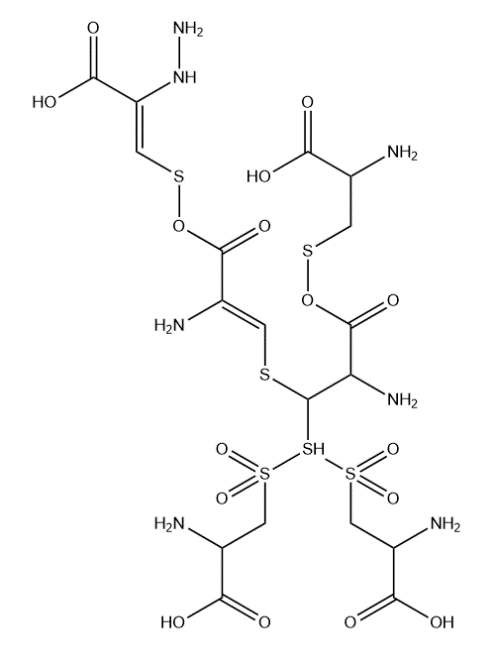  (2Z,7Z)-7,11,16-triamino-10-(1,3-bis(2-amino-2-carboxyethyl)-1,1,3,3-tetraoxo-1l6,2l4,3l6-trisulfaneyl)-2-hydrazineyl-6,12-dioxo-5,13-dioxa-4,9,14-trithiaheptadeca-2,7-dienedioic acid |
| 24 | C_20_H_35_N_7_O_16_S_6_ | 822.22 | 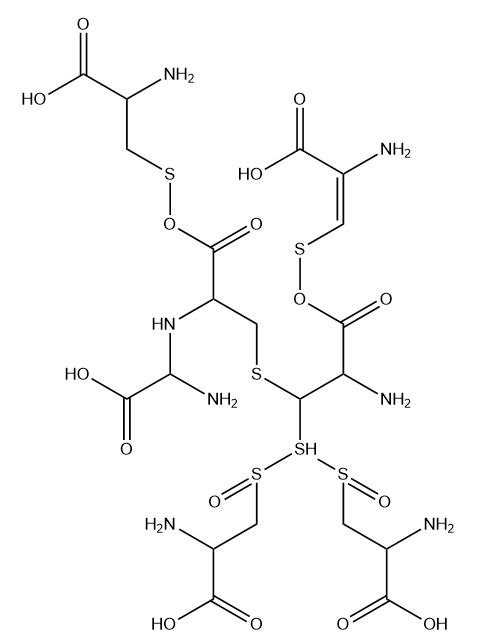  (E)-2,8,13-triamino-4-((((2-amino-2-carboxyethyl)thio)oxy)carbonyl)-7-(1,3-bis(2-amino-2-carboxyethyl)-1,3-dioxo-1l4,2l4,3l4-trisulfaneyl)-9-oxo-10-oxa-6,11-dithia-3-azatetradec-12-enedioic acid |
| 25 | C_18_H_36_N_8_O_18_S_7_ | 877.72 | 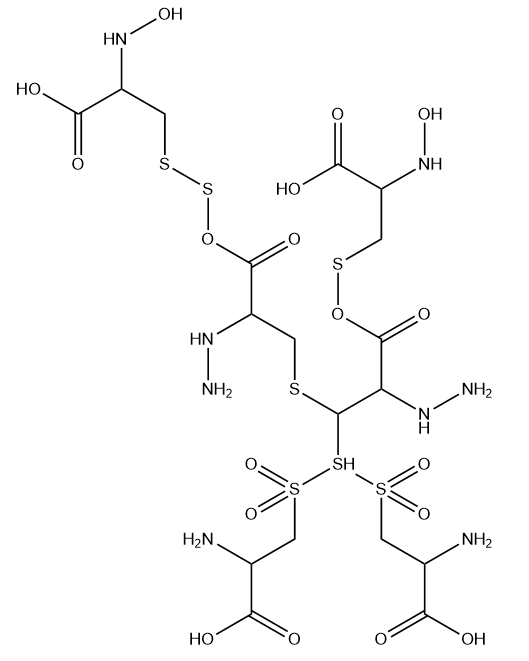  11-(1,3-bis(2-amino-2-carboxyethyl)-1,1,3,3-tetraoxo-1l6,2l4,3l6-trisulfaneyl)-8,12-dihydrazineyl-2,17-bis(hydroxyamino)-7,13-dioxo-6,14-dioxa-4,5,10,15-tetrathiaoctadecanedioic acid |
| 26 | C_23_H_36_N_6_O_19_S_7_ | 925.52 | 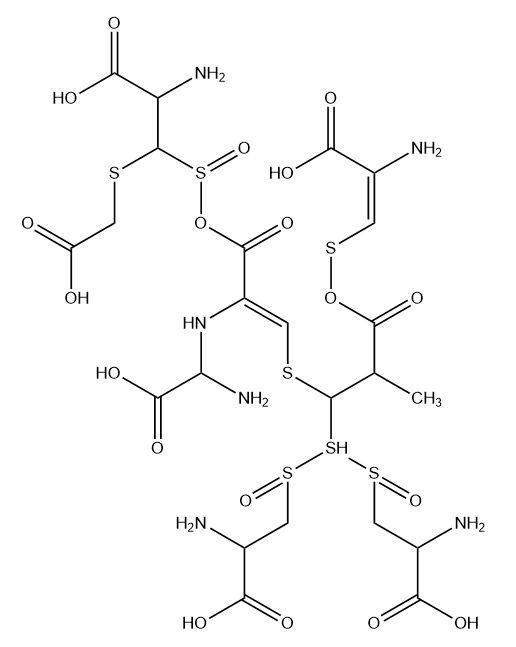  (4Z,12E)-2,13-diamino-4-((((2-amino-2-carboxy-1-((carboxymethyl)thio)ethyl)sulfinyl)oxy)carbonyl)-7-(1,3-bis(2-amino-2-carboxyethyl)-1,3-dioxo-1l4,2l4,3l4-trisulfaneyl)-8-methyl-9-oxo-10-oxa-6,11-dithia-3-azatetradeca-4,12-dienedioic acid |

**Table S2: Biomolecules derived from Ar+DMSO plasma**

|  | Formula | [M-H] ^+^(m/z) | Chemical Structure/  Systematic name |
| --- | --- | --- | --- |
| 1 | C_2_H_4_O_3_ | 77.92 | 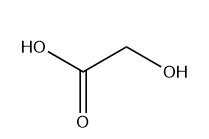  2-hydroxyacetic acid |
| 2 | CH_5_NO_2_S | 95.62 | 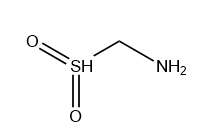  hydrosulfonylmethanamine |
| 3 | C_4_H_7_NO_2_ | 102.62 | 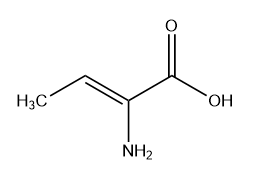  (Z)-2-aminobut-2-enoic acid |
| 4 | C_5_H_9_NO_2_ | 115.72 | 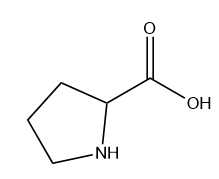  proline |
| 5 | C_3_H_7_NO_2_S | 122.02 |   cysteine |
| 6 | C_5_H_7_NO_3_ | 130.92 | 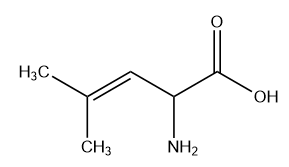  2-acetamidoacrylic acid |
| 7 | C_3_H_9_NO_3_S | 140.62 | 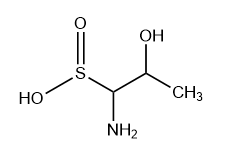  1-amino-2-hydroxypropane-1-sulfinic acid |
| 8 | C_3_H_11_NO_5_S | 174.02 | 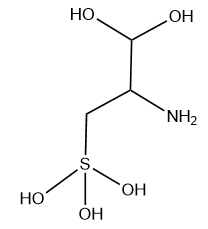  (2-amino-3,3-dihydroxypropyl)-l4-sulfanetriol |
| 9 | C_8_H_12_N_2_O_4_S_3_ | 297.12 | 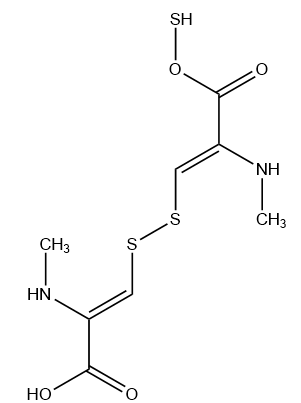  (Z)-3-(((Z)-3-mercaptooxy-2-(methylamino)-3-oxoprop-1-en-1-yl) disulfaneyl)-2-(methylamino)acrylic acid |
| 10 | C_6_H_13_N_3_O_9_S_2_ | 336.22 | 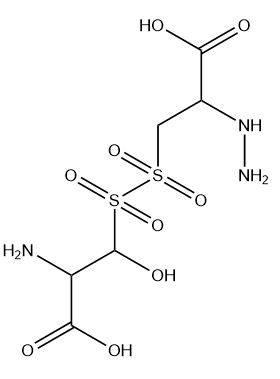  (((2-carboxy-2-hydrazineylethyl)sulfonyl)sulfonyl)serine |
| 11 | C_8_H_14_N_2_O_9_S_2_ | 347.52 | 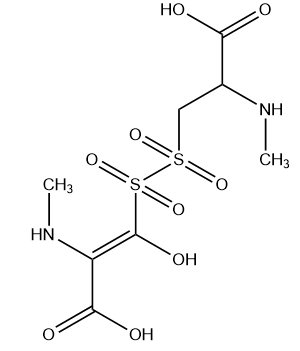  (Z)-3-(((2-carboxy-2-(methylamino)ethyl)sulfonyl)sulfonyl)-3-hydroxy-2-(methylamino)acrylic acid |
| 12 | C_10_H_18_N_2_O_8_S_3_ | 391.32 | 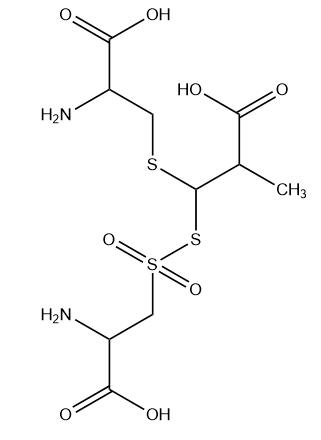  3-(((2-amino-2-carboxyethyl)sulfonyl)thio)-3-((2-amino-2-carboxyethyl)thio)-2-methylpropanoic acid |
| 13 | C_9_H_16_N_4_O_8_S_3_ | 405.22 | 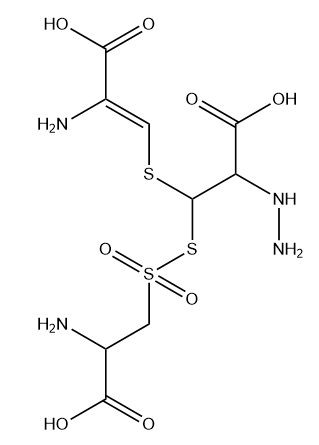  (Z)-2-amino-3-((1-(((2-amino-2-carboxyethyl)sulfonyl)thio)-2-carboxy-2-hydrazineylethyl)thio)acrylic acid |
| 14 | C_11_H_19_N_3_O_9_S_3_ | 434.72 | 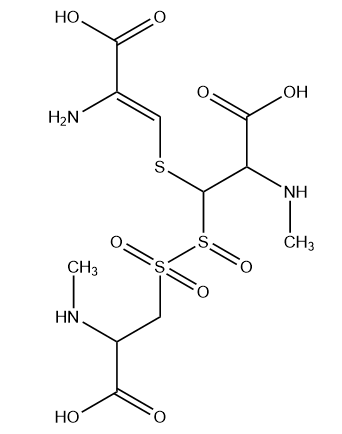  (Z)-2-amino-3-((2-carboxy-1-(((2-carboxy-2-(methylamino)ethyl)sulfonyl)sulfinyl)-2-(methylamino)ethyl)thio)acrylic acid |
| 15 | C_10_H_14_N_4_O_10_S_4_ | 478.92 | 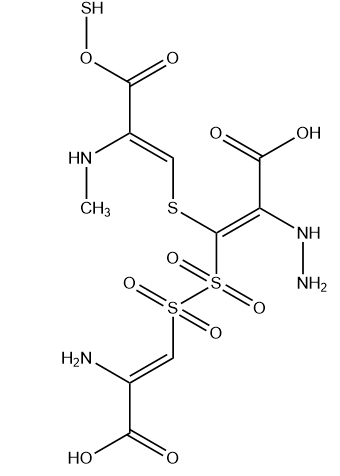  (Z)-3-((((Z)-2-amino-2-carboxyvinyl)sulfonyl)sulfonyl)-2-hydrazineyl-3-(((Z)-3-mercaptooxy-2-(methylamino)-3-oxoprop-1-en-1-yl)thio)acrylic acid |
| 16 | C_12_H_22_N_2_O_11_S_4_ | 499.82 | 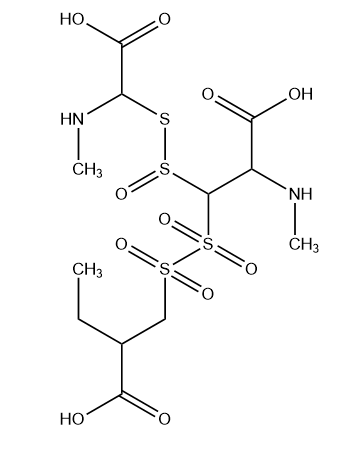  2-((((2-carboxy-1-(((carboxy(methylamino)methyl)thio)sulfinyl)-2-(methylamino)ethyl)sulfonyl)sulfonyl)methyl)butanoic acid |
| 17 | C_12_H_21_N_3_O_12_S_4_ | 528.02 | 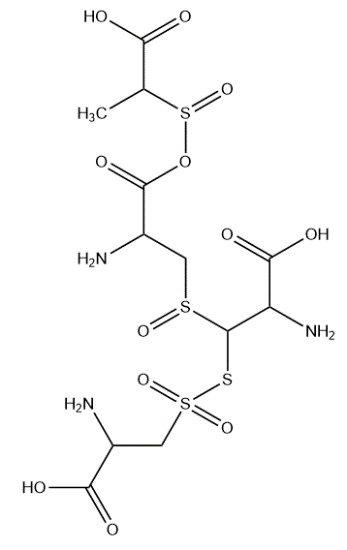  S-((2-amino-2-carboxyethyl)sulfonyl)-C3-((2-amino-3-(((1-carboxyethyl)sulfinyl)oxy)-3-oxopropyl)sulfinyl)cysteine |
| 18 | C_9_H_20_N_4_O_17_S_4_ | 584.12 | 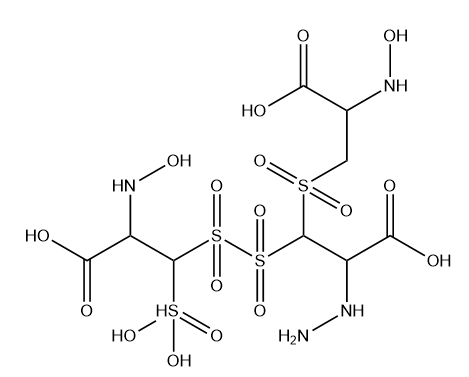  (((2-carboxy-1-((2-carboxy-2-(hydroxyamino)ethyl)sulfonyl)-2-hydrazineylethyl)sulfonyl)sulfonyl)(dihydroxy(oxo)-l6-sulfaneyl)(hydroxy)alanine |
| 19 | C_11_H_20_N_4_O_16_S_6_  C_11_H_16_N_2_O_18_S_6_ | 656.12 | 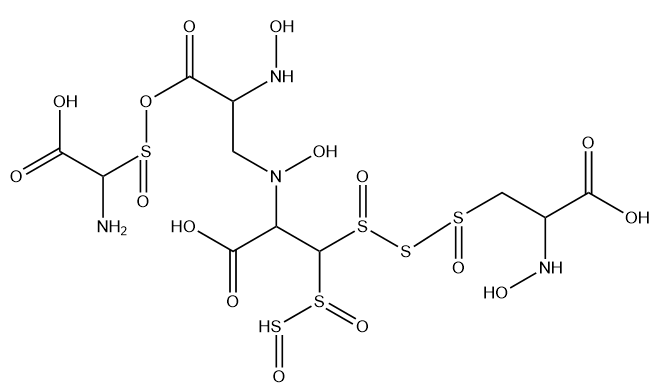  N-(3-(((amino(carboxy)methyl)sulfinyl)oxy)-2-(hydroxyamino)-3-oxopropyl)-C3-((((2-carboxy-2-(hydroxyamino)ethyl)sulfinyl)thio)sulfinyl)-C3-(hydrosulfinylsulfinyl)-N-hydroxyalanine  Or  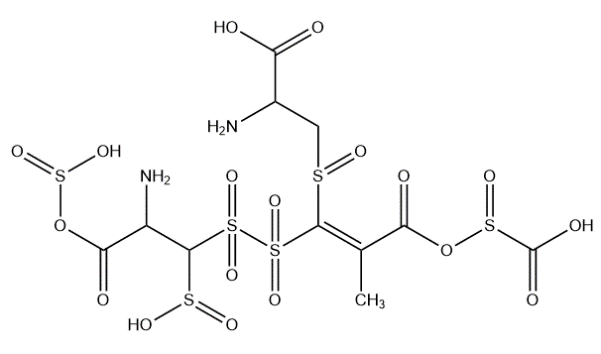  (E)-((1-(((2-amino-3-oxo-1-sulfino-3-(sulfinooxy)propyl)sulfonyl)sulfonyl)-3-((carboxysulfinyl)oxy)-2-methyl-3-oxoprop-1-en-1-yl)sulfinyl)alanine |
| 20 | C_18_H_29_N_5_O_15_S_6_ | 749.42 | 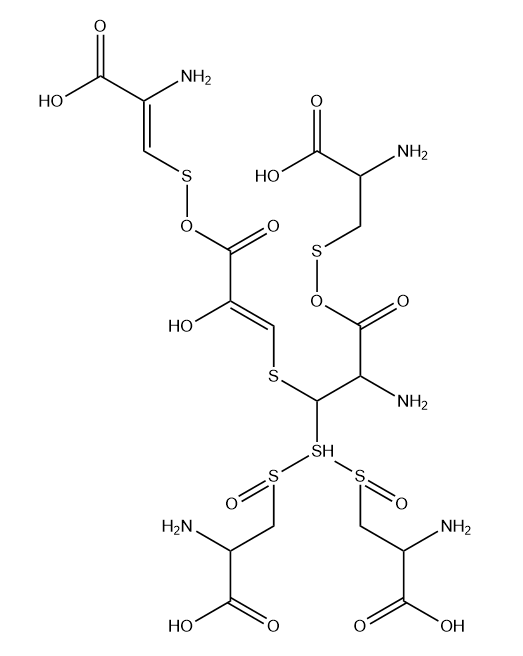  (2Z,7Z)-2,11,16-triamino-10-(1,3-bis(2-amino-2-carboxyethyl)-1,3-dioxo-1l4,2l4,3l4-trisulfaneyl)-7-hydroxy-6,12-dioxo-5,13-dioxa-4,9,14-trithiaheptadeca-2,7-dienedioic acid |
| 21 | C_18_H_31_N_7_O_16_S_6_ | 794.52 | 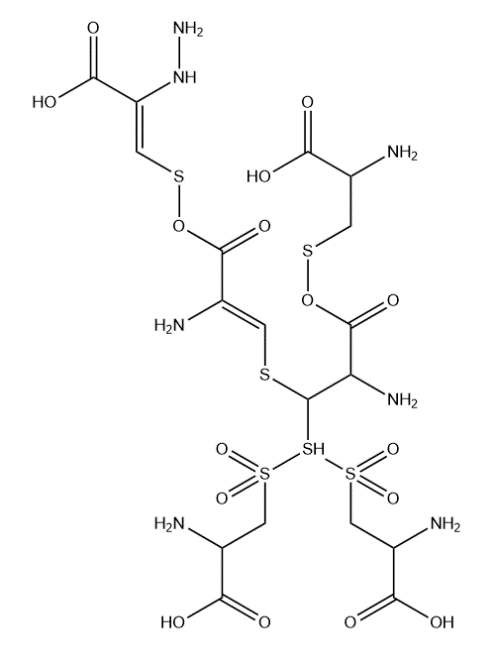  (2Z,7Z)-7,11,16-triamino-10-(1,3-bis(2-amino-2-carboxyethyl)-1,1,3,3-tetraoxo-1l6,2l4,3l6-trisulfaneyl)-2-hydrazineyl-6,12-dioxo-5,13-dioxa-4,9,14-trithiaheptadeca-2,7-dienedioic acid |
| 22 | C_18_H_35_N_7_O_18_S_7_ | 862.62 | 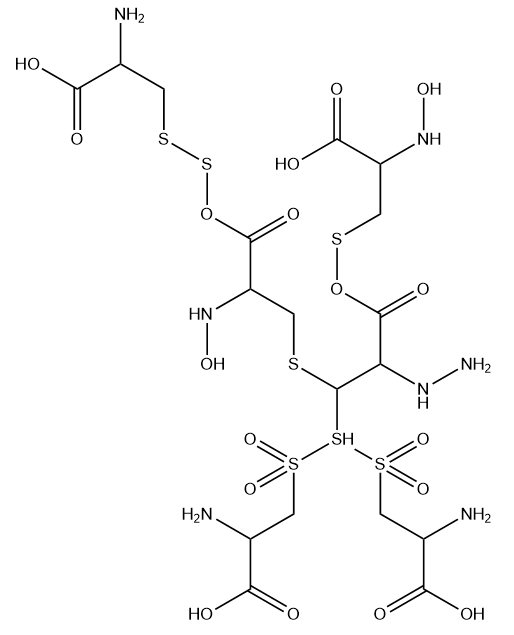  2-amino-11-(1,3-bis(2-amino-2-carboxyethyl)-1,1,3,3-tetraoxo-1l6,2l4,3l6-trisulfaneyl)-12-hydrazineyl-8,17-bis(hydroxyamino)-7,13-dioxo-6,14-dioxa-4,5,10,15-tetrathiaoctadecanedioic acid |
| 23 | C_23_H_33_N_7_O_19_S_7_  C_23_H_33_N_7_O_17_S_8_ | 936.82 | 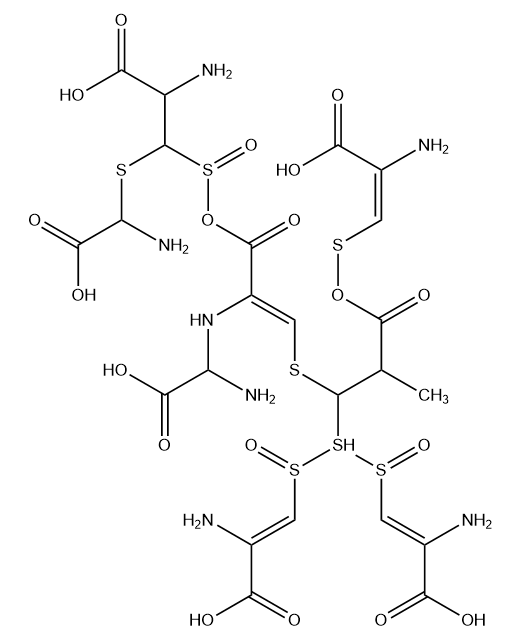  (4Z,12E)-2,13-diamino-4-((((2-amino-1-((amino(carboxy)methyl)thio)-2-carboxyethyl)sulfinyl)oxy)carbonyl)-7-(1,3-bis((Z)-2-amino-2-carboxyvinyl)-1,3-dioxo-1l4,2l4,3l4-trisulfaneyl)-8-methyl-9-oxo-10-oxa-6,11-dithia-3-azatetradeca-4,12-dienedioic acid  Or  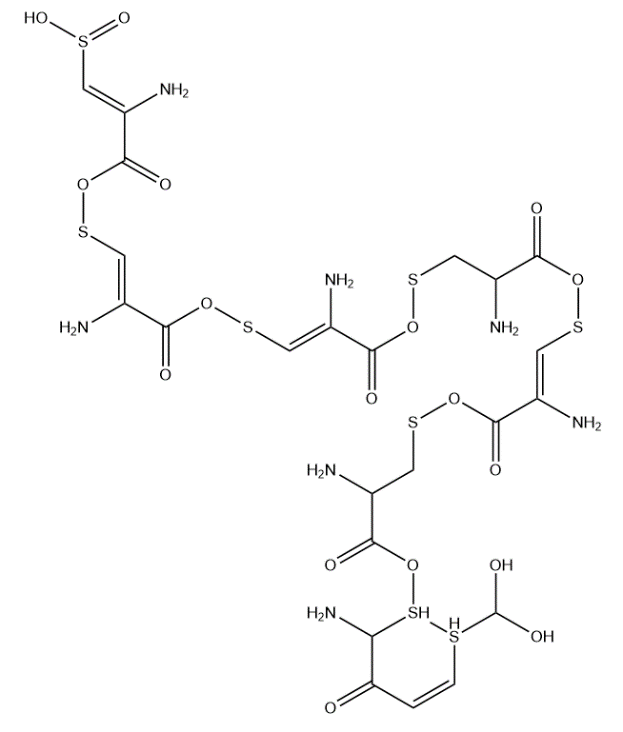  (1Z,6Z,11Z,21Z)-2,7,12,17,22,27-hexaamino-28-((3-amino-1-(dihydroxymethyl)-4-oxo-3,4-dihydro-1H-1l4,2l4-dithiin-2-yl)oxy)-3,8,13,18,23,28-hexaoxo-4,9,14,19,24-pentaoxa-5,10,15,20,25-pentathiaoctacosa-1,6,11,21-tetraene-1-sulfinic acid |

**Table S3: Biomolecules derived from Ar+H_2_O_2_+DMSO plasma**

|  | Formula | [M-H] ^+^(m/z) | Chemical Structure/  Systematic name |
| --- | --- | --- | --- |
| 1 | C_2_H_4_O_3_ | 77.52 | 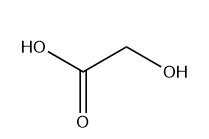  2-hydroxyacetic acid |
| 2 | CH_3_NO_2_S | 93.92 | 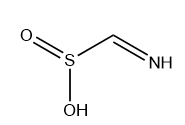  iminomethanesulfinic acid |
| 3 | C_4_H_7_NO_2_ | 102.82 | 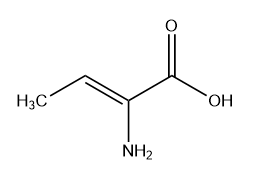  (Z)-2-aminobut-2-enoic acid |
| 4 | C_5_H_7_NO_2_ | 114.02 | 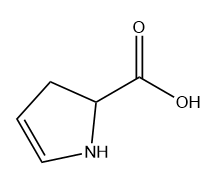  2,3-dihydro-1H-pyrrole-2-carboxylic acid |
| 5 | C_3_H_7_NO_2_S | 122.02 |   cysteine |
| 6 | C_7_H_14_O_2_ | 131.02 | 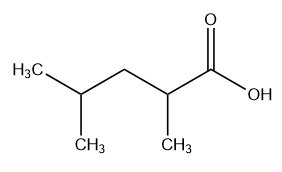  2,4-dimethylpentanoic acid |
| 7 | C_3_H_8_O_4_S | 141.02 | 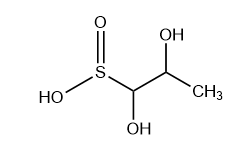  1,2-dihydroxypropane-1-sulfinic acid |
| 8 | C_5_H_9_NO_4_ | 148.72 | 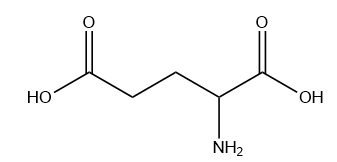  glutamic acid |
| 9 | C_3_H_11_NO_5_S | 174.32 | 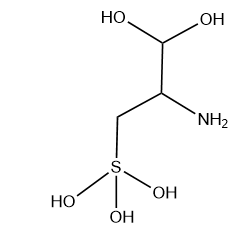  (2-amino-3,3-dihydroxypropyl)-l4-sulfanetriol |
| 10 | C_4_H_9_NO_3_S_2_  C_4_H_9_NO_3_S_2_ | 184.12 | 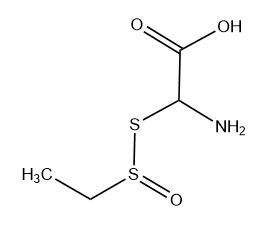  2-amino-2-((ethylsulfinyl)thio)acetic acid  Or  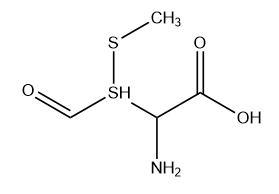  C2-(1-formyl-2-methyl-1l4-disulfaneyl)glycine |
| 11 | C_6_H_16_N_2_O_9_S_2_  C_9_H_11_NO_6_S_3_ | 325.82 | 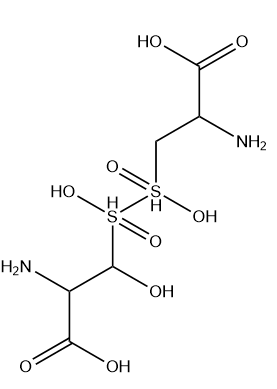  (2-(2-amino-2-carboxyethyl)-1,2-dihydroxy-1,2-dioxo-1l6,2l6-disulfaneyl)serine  Or  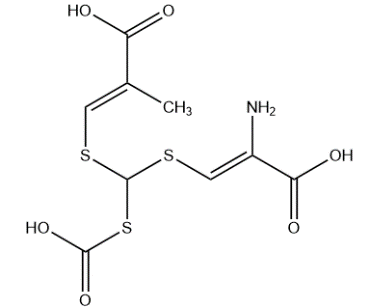  (Z)-2-amino-3-(((((E)-2-carboxyprop-1-en-1-yl)thio)(carboxythio)methyl)thio)acrylic acid |
| 12 | C_6_H_13_N_3_O_9_S_2_ | 336.32 | 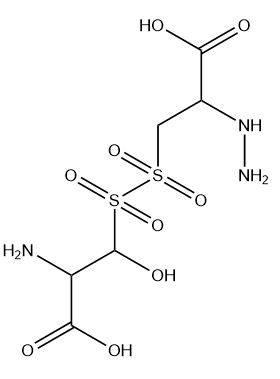  (((2-carboxy-2-hydrazineylethyl)sulfonyl)sulfonyl)serine |
| 13 | C_8_H_14_N_2_O_9_S_2_ | 347.52 | 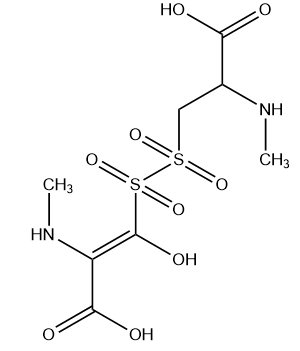  (Z)-3-(((2-carboxy-2-(methylamino)ethyl)sulfonyl)sulfonyl)-3-hydroxy-2-(methylamino)acrylic acid |
| 14 | C_9_H_16_N_2_O_8_S_3_ | 377.32 | 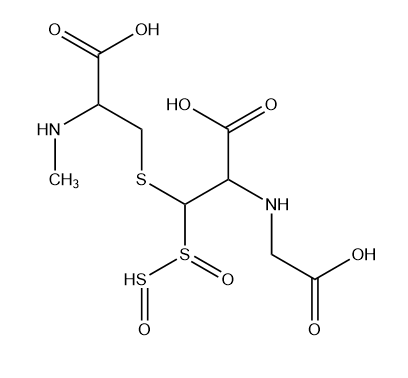  S-(2-carboxy-2-(methylamino)ethyl)-N-(carboxymethyl)-C3-(hydrosulfinylsulfinyl)cysteine |
| 15 | C_9_H_16_N_4_O_8_S_3_ | 405.42 | 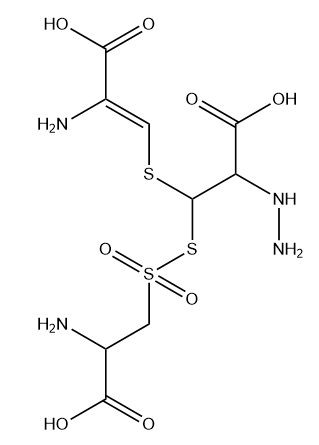  (Z)-2-amino-3-((1-(((2-amino-2-carboxyethyl)sulfonyl)thio)-2-carboxy-2-hydrazineylethyl)thio)acrylic acid |
| 16 | C_11_H_17_N_3_O_8_S_3_ | 416.92 | 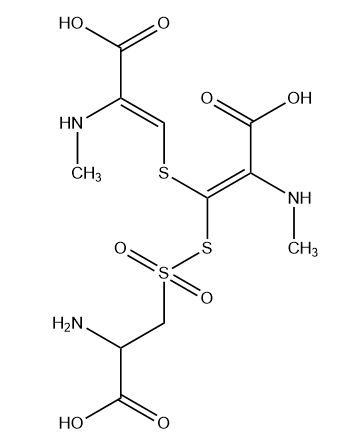  (Z)-3-(((2-amino-2-carboxyethyl)sulfonyl)thio)-3-(((Z)-2-carboxy-2-(methylamino)vinyl)thio)-2-(methylamino)acrylic acid |
| 17 | C_9_H_14_N_4_O_10_S_3_ | 434.82 | 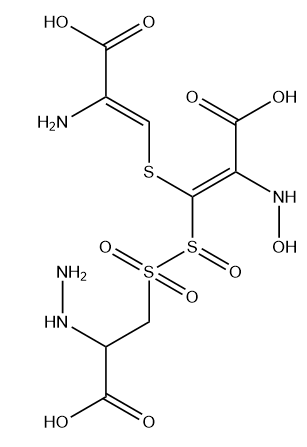  (Z)-3-(((Z)-2-amino-2-carboxyvinyl)thio)-3-(((2-carboxy-2-hydrazineylethyl)sulfonyl)sulfinyl)-2-(hydroxyamino)acrylic acid |
| 18 | C_9_H_13_N_5_O_10_S_3_ | 448.52 | 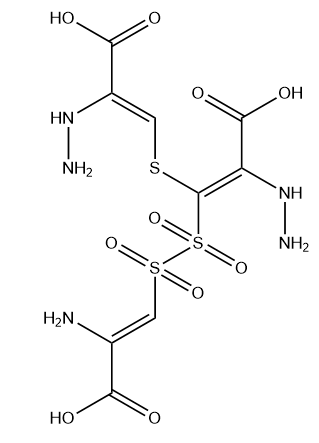  (Z)-3-((((Z)-2-amino-2-carboxyvinyl)sulfonyl)sulfonyl)-3-(((Z)-2-carboxy-2-hydrazineylvinyl)thio)-2-hydrazineylacrylic acid |
| 19 | C_10_H_14_N_4_O_10_S_4_ | 478.72 | 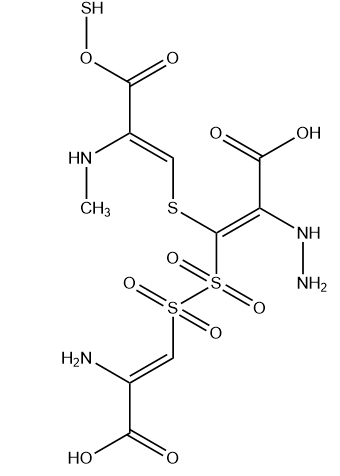  (Z)-3-((((Z)-2-amino-2-carboxyvinyl)sulfonyl)sulfonyl)-2-hydrazineyl-3-(((Z)-3-mercaptooxy-2-(methylamino)-3-oxoprop-1-en-1-yl)thio)acrylic acid |
| 20 | C_12_H_21_N_3_O_12_S_4_ | 528.32 | 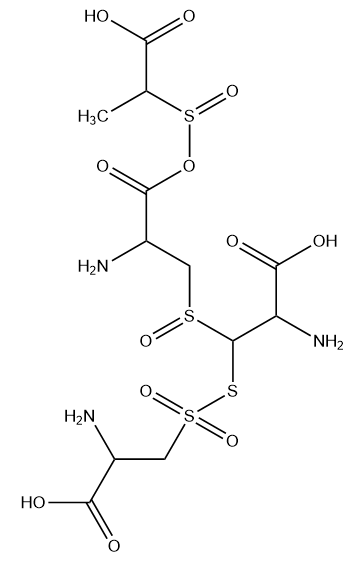  S-((2-amino-2-carboxyethyl)sulfonyl)-C3-((2-amino-3-(((1-carboxyethyl)sulfinyl)oxy)-3-oxopropyl)sulfinyl)cysteine |
| 21 | C_9_H_21_N_5_O_16_S_4_ | 583.52 | 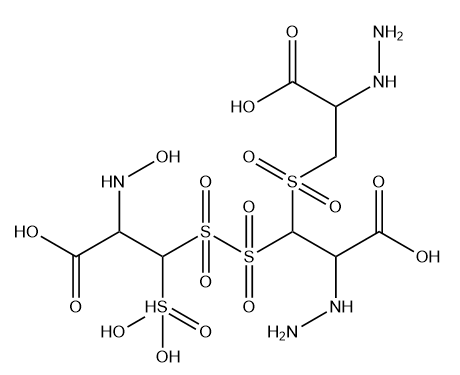  (((2-carboxy-1-((2-carboxy-2-hydrazineylethyl)sulfonyl)-2-hydrazineylethyl)sulfonyl)sulfonyl)(dihydroxy(oxo)-l6-sulfaneyl)(hydroxy)alanine |
| 22 | C_9_H_16_N_6_O_17_S_4_ | 608.42 | 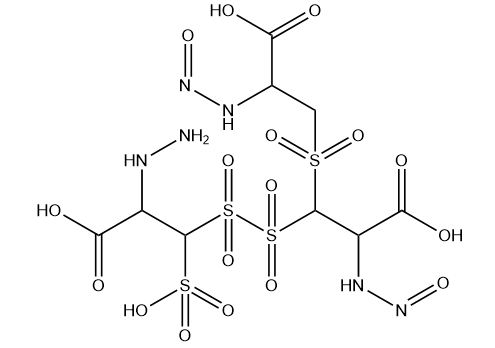  amino(((2-carboxy-1-((2-carboxy-2-(nitrosoamino)ethyl)sulfonyl)-2-(nitrosoamino)ethyl)sulfonyl)sulfonyl)(sulfo)alanine |
| 23 | C_11_H_20_N_4_O_16_S_6_  C_11_H_16_N_2_O_18_S_6_ | 656.02 | 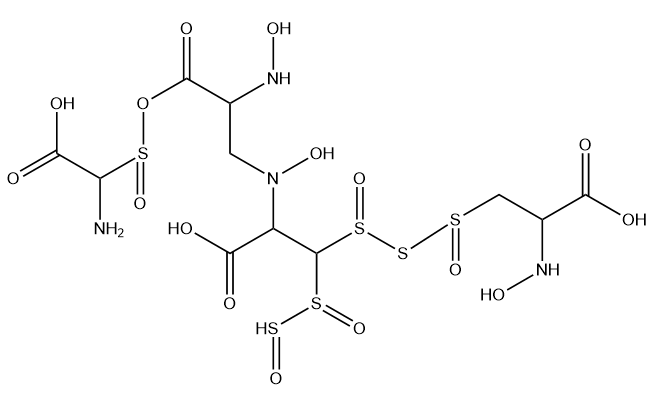  N-(3-(((amino(carboxy)methyl)sulfinyl)oxy)-2-(hydroxyamino)-3-oxopropyl)-C3-((((2-carboxy-2-(hydroxyamino)ethyl)sulfinyl)thio)sulfinyl)-C3-(hydrosulfinylsulfinyl)-N-hydroxyalanine  Or  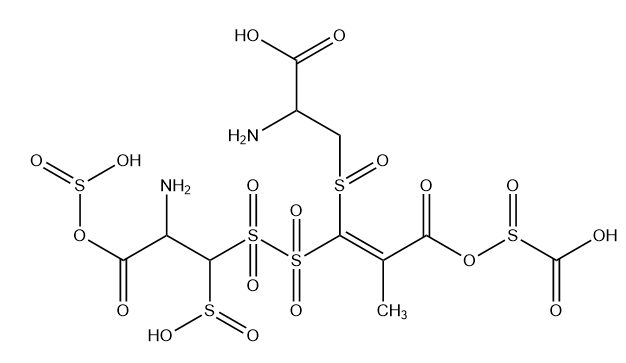  (E)-((1-(((2-amino-3-oxo-1-sulfino-3-(sulfinooxy)propyl)sulfonyl)sulfonyl)-3-((carboxysulfinyl)oxy)-2-methyl-3-oxoprop-1-en-1-yl)sulfinyl)alanine |
| 24 | C_24_H_36_O_16_S_6_ | 773.42 | 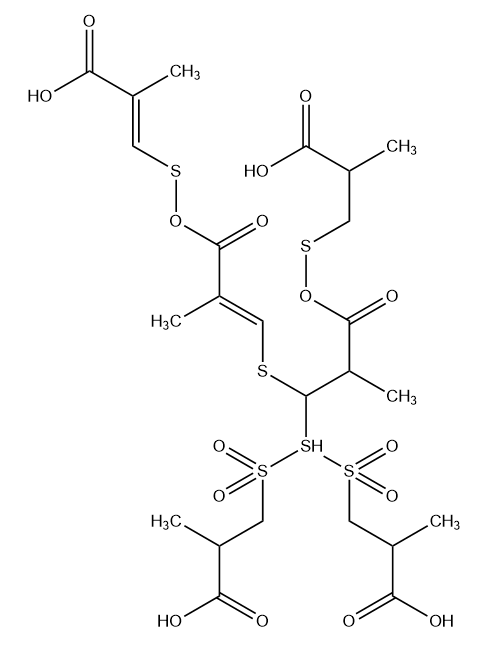  (2E,7E)-10-(1,3-bis(2-carboxypropyl)-1,1,3,3-tetraoxo-1l6,2l4,3l6-trisulfaneyl)-2,7,11,16-tetramethyl-6,12-dioxo-5,13-dioxa-4,9,14-trithiaheptadeca-2,7-dienedioic acid |
| 25 | C_18_H_31_N_7_O_16_S_6_ | 794.82 | 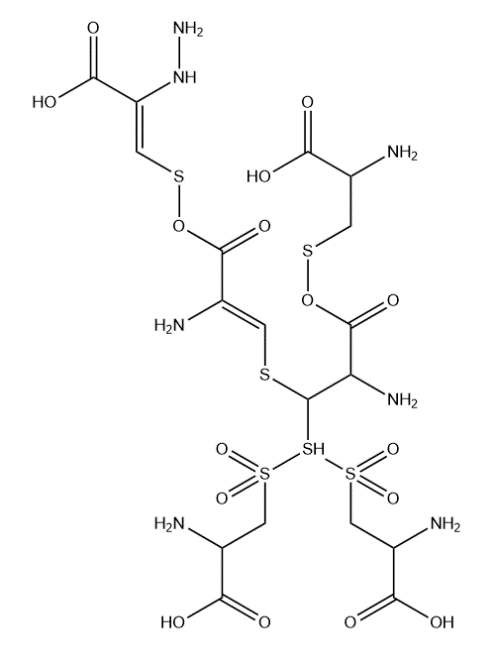  (2Z,7Z)-7,11,16-triamino-10-(1,3-bis(2-amino-2-carboxyethyl)-1,1,3,3-tetraoxo-1l6,2l4,3l6-trisulfaneyl)-2-hydrazineyl-6,12-dioxo-5,13-dioxa-4,9,14-trithiaheptadeca-2,7-dienedioic acid |
| 26 | C_18_H_35_N_7_O_18_S_7_ | 862.32 | 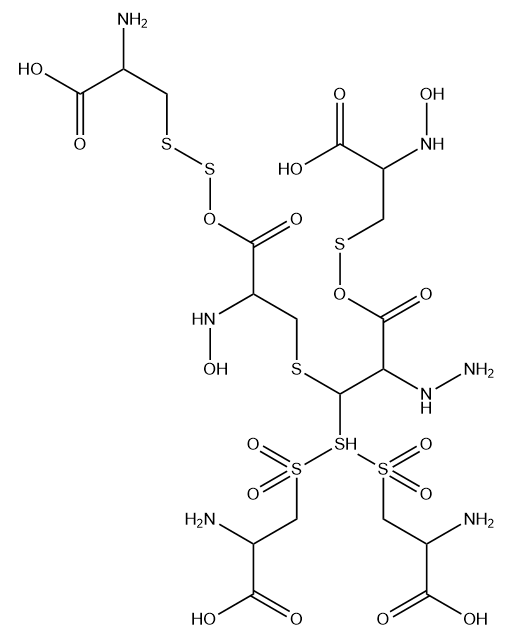  2-amino-11-(1,3-bis(2-amino-2-carboxyethyl)-1,1,3,3-tetraoxo-1l6,2l4,3l6-trisulfaneyl)-12-hydrazineyl-8,17-bis(hydroxyamino)-7,13-dioxo-6,14-dioxa-4,5,10,15-tetrathiaoctadecanedioic acid |
| 27 | C_24_H_37_N_5_O_19_S_7_ | 924.72 | 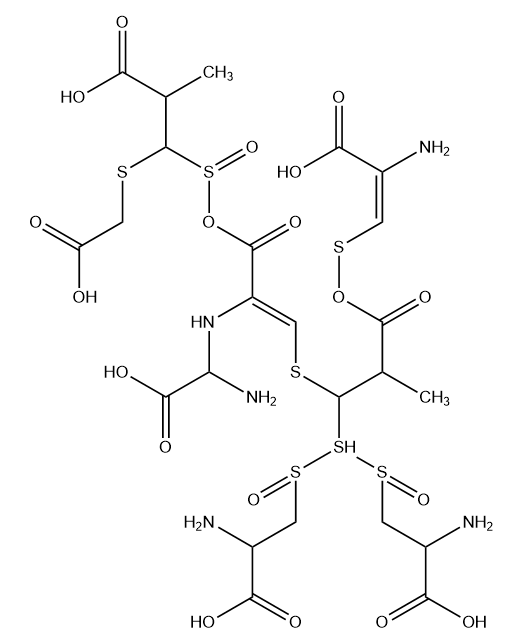  (4Z,12E)-2,13-diamino-7-(1,3-bis(2-amino-2-carboxyethyl)-1,3-dioxo-1l4,2l4,3l4-trisulfaneyl)-4-((((2-carboxy-1-((carboxymethyl)thio)propyl)sulfinyl)oxy)carbonyl)-8-methyl-9-oxo-10-oxa-6,11-dithia-3-azatetradeca-4,12-dienedioic acid |
| 28 | C_25_H_38_N_8_O_15_S_8_  C_24_H_33_N_7_O_19_S_7_ | 947.42 | 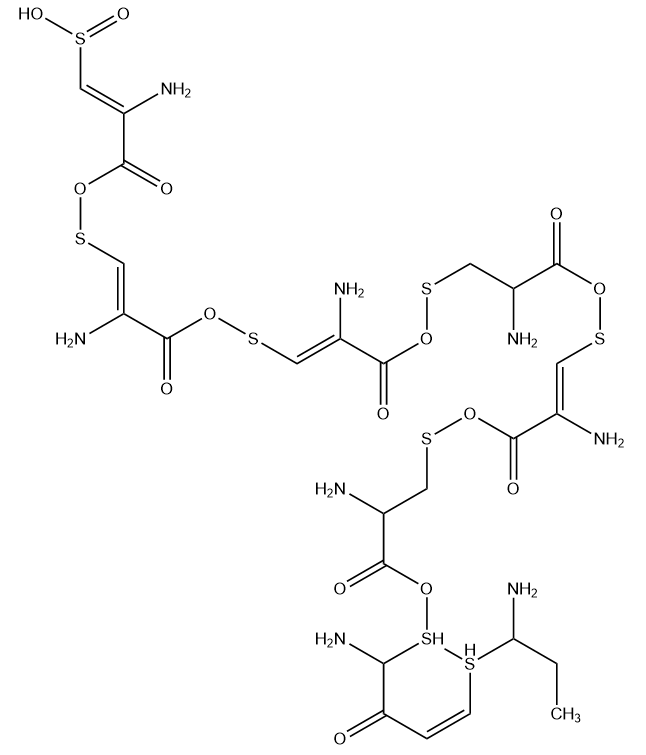  (1Z,6Z,11Z,21Z)-2,7,12,17,22,27-hexaamino-28-((3-amino-1-(1-aminopropyl)-4-oxo-3,4-dihydro-1H-1l4,2l4-dithiin-2-yl)oxy)-3,8,13,18,23,28-hexaoxo-4,9,14,19,24-pentaoxa-5,10,15,20,25-pentathiaoctacosa-1,6,11,21-tetraene-1-sulfinic acid  Or  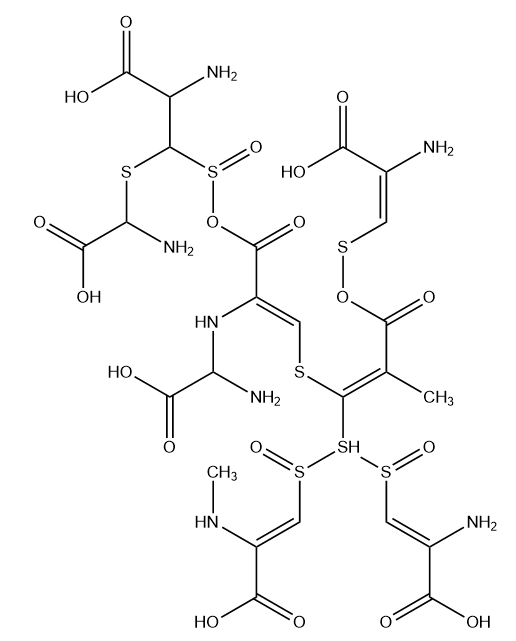  (4Z,7E,12E)-2,13-diamino-4-((((2-amino-1-((amino(carboxy)methyl)thio)-2-carboxyethyl)sulfinyl)oxy)carbonyl)-7-(1-((Z)-2-amino-2-carboxyvinyl)-3-((Z)-2-carboxy-2-(methylamino)vinyl)-1,3-dioxo-1l4,2l4,3l4-trisulfaneyl)-8-methyl-9-oxo-10-oxa-6,11-dithia-3-azatetradeca-4,7,12-trienedioic acid |
